# Effectiveness of the Assessment of Burden of Chronic Conditions (ABCC)-tool in patients with asthma, COPD, type 2 diabetes mellitus, and heart failure: A pragmatic clustered quasi-experimental study in the Netherlands

**DOI:** 10.1080/13814788.2024.2343364

**Published:** 2024-05-13

**Authors:** Esther A. Boudewijns, Danny Claessens, Onno C.P van Schayck, Mascha Twellaar, Bjorn Winkens, Manuela A. Joore, Lotte C. E. M Keijsers, Stijn Krol, Mathijs Urlings, Annerika H. M Gidding-Slok

**Affiliations:** a Department of Family Medicine, Care and Public Health Research Institute (CAPHRI), Maastricht University, Maastricht, the Netherlands; b Department of Methodology and Statistics, Care and Public Health Research Institute (CAPHRI), Maastricht University, Maastricht, the Netherlands; c Department of Clinical Epidemiology and Medical Technology Assessment (KEMTA), Maastricht University Medical Centre MUMC+/Care and Public Health Research Institute (CAPHRI), Maastricht University, Maastricht, the Netherlands

**Keywords:** Chronic conditions, person-centred care, self-management, shared decision making, general practice

## Abstract

**Background:**

The Assessment of Burden of Chronic Conditions (ABCC)-tool was developed to optimise chronic care.

**Objectives:**

This study aimed to assess the effectiveness of the ABCC-tool in patients with COPD, asthma, type 2 diabetes, and/or heart failure in primary care in the Netherlands.

**Methods:**

The study had a pragmatic, clustered, two-armed, quasi-experimental design. The intervention group (41 general practices; 176 patients) used the ABCC-tool during routine consultations and the control group (14 general practices; 61 patients) received usual care. The primary outcome was a change in perceived quality of care (PACIC; Patient Assessment of Chronic Illness Care) after 18 months. Secondary outcomes included change in the PACIC after 6 and 12 months, and in quality of life (EQ-5D-5L; EuroQol-5D-5L), capability well-being (ICECAP-A; ICEpop CAPability measure for Adults), and patients’ activation (PAM; Patient Activation Measure) after 6, 12, and 18 months for the total group and conditions separately.

**Results:**

We observed a significant difference in the PACIC after 6, 12, and 18 months (18 months: 0.388 points; 95%CI: 0.089–0.687; *p* = 0.011) for the total group and after 6 and 12 months for type 2 diabetes. After 18 months, we observed a significant difference in the PAM for the total group but not at 6 and 12 months, and not for type 2 diabetes. All significant effects were in favour of the intervention group. No significant differences were found for the EQ-5D-5L and the ICECAP-A.

**Conclusion:**

Use of the ABCC-tool has a positive effect on perceived quality of care and patients’ activation, which makes the tool ready for use in clinical practice. Healthcare providers (e.g. general practitioners and practice nurses) can use the tool to provide person-centred care.

**Trial registration number:** ClinicalTrials.gov Registry (NCT04127383).

## KEY MESSAGES

The Assessment of Burden of Chronic Conditions (ABCC)-tool aims to support disease management for one or multiple chronic condition(s), currently COPD, asthma, type 2 diabetes, and heart failure.

Statistically significant differences in patients’ perceived quality of care and patient activation were found between the group that used the ABCC-tool and the care-as-usual group. No effect was found on generic quality of life or capability well-being.

Healthcare providers can use the ABCC-tool in primary care.

## Introduction

**Figure 1.**
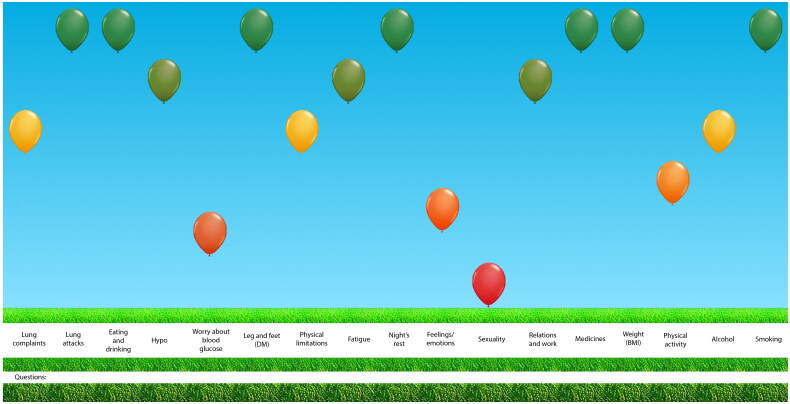
Visualisation of the ABCC-tool for a person with COPD and type 2 diabetes.

The increasing number of people with chronic conditions represents one of the principal challenges for healthcare systems today [1, 2]. This is also the case for primary care in the Netherlands, as most care for people with chronic conditions is provided in general practice [3]. It has stressed the need for a transformation of the healthcare system for chronic conditions from one with largely passive participation of patients to one in which people are expected to actively participate in managing their own health [1, 2]. As this requires knowledge, confidence, and skills, care for people with chronic conditions should be person-centred and focused on strategies to engage, support, and empower them [4–6]. The Assessment of Burden of Chronic Conditions (ABCC)-tool was developed to aid healthcare providers, such as general practitioners (GPs), practice nurses, and nurse practitioners, in providing a personalised approach to care for people with one or more chronic condition(s). The tool is developed based on the principles of shared decision-making and supports self-management. The tool consists of a patient reported outcome measure (PROM), a visualisation of the results, and treatment advice to integrate this in a conversation about a personalised care plan (Box 1 and Figure 1). It is currently developed for people with Chronic Obstructive Pulmonary Disease (COPD), asthma, type 2 diabetes, and heart failure. A full description of the ABCC-tool, including its questions and domains, is published elsewhere [7]. A module has been developed for each condition. Per patient the appropriate modules can be selected. The different modules are combined into one PROM and one visual overview. The ABCC-tool allows the combination of disease management programmes for multiple chronic conditions for one person, facilitating personalised care. The current study builds on the evidence base surrounding the Assessment of Burden of COPD-tool, the predecessor of the ABCC-tool, which has shown to be effective in improving quality of life and perceived quality of care [8–10]. The primary aim of the current study was to assess the effectiveness of using the ABCC-tool in patients with COPD, asthma, type 2 diabetes, or heart failure (or a combination of these) on the perceived quality of care, as measured by the Patient Assessment of Chronic Illness Care (PACIC), after 18 months as compared to usual care.

## Methods

### Study design

The study had a pragmatic, clustered, two-armed, quasi-experimental design. It was conducted in 55 general practices across the Netherlands from November 2019 to November 2022, with a follow-up period of 18 months. Although not every patient had completed the follow-up time by November 2022, we had to stop collecting data for feasibility reasons (i.e. considering our time and budget constraints). The trial was registered at ClinicalTrials.gov Registry (NCT04127383). A detailed protocol for this study has been published elsewhere [11]. Deviations from the protocol are described in Supplementary Material 1. We followed the Transparent Reporting of Evaluations with Nonrandomised Designs (TREND) reporting standard [12]. Supplementary Material 2 provides more information on design choices using the Pragmatic Explanatory Continuum Indicator Summary-2 (PRECIS-2) tool [13].

### Participants, recruitment, and allocation

The intervention was allocated at a general practice level to avoid contamination bias. The researchers recruited general practices. One or more healthcare provider(s) could participate within a general practice. These included general practitioners, general practice nurses, and nurse practitioners. General practice nurses collaborate closely with the general practitioner and are concerned with prevention, monitoring, guidance, information and education for patients, mainly people with chronic conditions. Nurse practitioners can independently take over tasks from a general practitioner and guide patients with highly complex care needs.

In the Netherlands, there are several information systems. The ABCC-tool was implemented in two of these information systems: a GP information system and a chain care information system. We excluded general practices that used the Assessment of Burden of COPD (ABC)-tool [8], the predecessor of the ABCC-tool, from the control group to avoid contamination bias. Consequently, if we had used randomisation, general practices should have had access to the ABCC-tool (a requirement for the intervention group) and should not have used the ABC-tool (a requirement for the control group). This would have resulted in a limited number of eligible general practices. Therefore, we conducted a quasi-experimental study in which we did not randomise general practices. We have decided not to randomise to ensure the feasibility of the study. Recruiting enough healthcare providers is a challenge, and with the limitations we had (see above) the likelihood that we would be unable to include enough healthcare providers was too high. Therefore, general practices that had the information systems in which the ABCC-tool was implemented, were allocated to the intervention group and general practices without access – and that had not used the ABC-tool previously – were allocated to the control group. General practices subsequently recruited patients. Eligibility criteria for patients were a diagnosis of COPD, asthma, type 2 diabetes and/or heart failure, aged over 18 years, and being able to understand and read the Dutch language. Exclusion criteria were the use of prednisolone due to exacerbations for people with asthma and COPD, and a hospitalisation for people with type 2 diabetes and heart failure within a 6-week period prior to the start of the study [11].

### Intervention and control group

Healthcare providers in the intervention group were instructed to use the ABCC-tool during routine consultations with their patients (Box 1 and Supplementary Material 3). Healthcare providers in the control group were instructed to provide usual care. Blinding of healthcare providers and patients was not possible.

### Measurements

Outcomes were measured at a patient’s level using self-reported questionnaires, completed online or via post. The questionnaire within the ABCC-tool was not used to measure outcomes. Outcomes were measured at four different points: at baseline, 6, 12, and 18 months. The questionnaires included the PACIC, EuroQol-5D-5L (EQ-5D-5L), Patient Activation Measure (PAM), and ICEpop (Investigating Choice Experiments for the Preferences of Older People) CAPability measure for Adults (ICECAP-A) (Supplementary Material 4). The PACIC has a five-point response scale, ranging from 1 = ‘almost never’ to 5 = ‘almost always.’ Higher scores mean more frequent presence of structured chronic care aspects [14]. The ICECAP-A measures capability well-being, defined as individuals’ ability to ‘be’ and ‘do’ the important things in life [15]. Healthcare providers completed a short questionnaire at baseline about their general practice. Furthermore, we collected data on a patient level regarding using the ABCC-tool from the information systems to assess intervention compliance.

### Outcomes

The primary outcome was a change from baseline in perceived quality of care, as measured by the PACIC, compared with usual care after 18 months for the total group. We hypothesised that the use of the ABCC-tool would result in significant improvements in the perceived quality of care compared to the control group after 18 months. Secondary outcomes included change in the PACIC subdomains after 18 months for the total group, in the PACIC (total score and subdomains) after 18 months for chronic conditions separately, in the PACIC (total score and subdomains) after 6 and 12 months for the total group and chronic conditions separately, and in quality of life (EQ-5D-5L), capability well-being (ICECAP-A), and patients’ activation (PAM) after 6, 12, and 18 months for the total group and chronic conditions separately. Due to the small number of patients with COPD, asthma, and heart failure (35, 28, and 30 patients at baseline respectively), we decided to not conduct separate analyses for each condition. Analyses were therefore only conducted for the total group (*n* = 235) and for type 2 diabetes (*n* = 197).

### Sample size

The sample size calculation indicated that 360 patients (180 patients per arm) from 36 general practices (18 general practices per arm) should be included. Regarding the primary outcome, PACIC, little is known about the minimal important difference and SD in the Dutch population. A medium effect size of 0.51 was estimated based on the study of Slok et al. [8]. Based on an independent-samples t-test, a significance level α of 0.05 for two-sided testing, a power (1 − β) of 90% and an intervention: control ratio of 1:1, 82 patients must be included per arm. To account for the design effect (= 1+(*m* − 1)*ICC, assuming an intraclass correlation (ICC) of 0.05 and a mean number of patients per general practice (m) of 10)[8] and for unequal cluster sizes (divided by 0.9)[16] and dropout of 25% (divided by 0.75), 177 participants per arm are required. So, 18 general practices (180 participants) must be included per arm.

A detailed description can be found in the protocol [11].

### Statistical analyses

Data were analysed according to the intention-to-treat principle. Multiple imputation was used to correct for missing data at baseline but not for outcomes (imputation model would become too large). Missing values were imputed using the fully conditional specification method, where predictive mean matching was used for numerical variables and logistic regression for categorical ones. Twenty iterations were used and 50 complete datasets were created. Outcomes were analysed using linear mixed models with a random intercept (and/or slope) on general practice level and an unstructured covariance structure for repeated measures (syntax in Supplementary Material 5). This resulted in a multilevel analysis with three levels: general practice, patient, and measurement. The fixed part consisted of ‘treatment arm,’ ‘time’ (categorical), and ‘treatment arm by time interaction.’ All potential confounders were included in our main analysis: national background (Dutch background/first-generation migrants/second-generation migrants); educational level (low/middle/high); age (years; continuous); sex (man/woman); body mass index (kg/m2; continuous); smoking status (never/former/current); asthma (yes/no); COPD (yes/no); type 2 diabetes (yes/no); heart failure (yes/no); other diseases (yes/no); location of the general practice (urban/rural); general practitioner with specialisation in COPD, asthma, type 2 diabetes, or heart failure (yes/no); year of graduation managing general practitioner (10 years ago); general practice in a health centre that is, availability of pharmacy, psychologist or physiotherapist in the same building (yes/no); the possibility of consulting a specialist within primary care (yes/no), and diagnosed COVID-19 during the study period (yes/no). Adjusted treatment effects were reported with corresponding 95% confidence intervals and *P*-values. A *P*-value of ≤ 0.05 was considered statistically significant. All analyses were performed using IBM SPSS Statistics, Version 27. The researchers were not blinded. Information about sensitivity analyses is provided in Supplementary Material 6.

## Results

**Figure 2.**
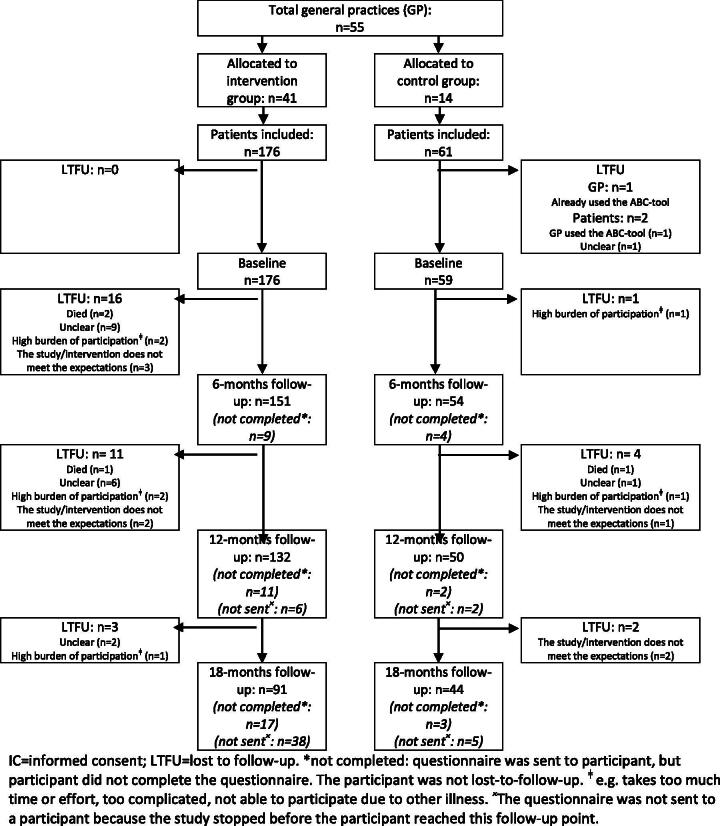
Flowchart of patients in the study. IC = informed consent; LTFU = lost to follow-up. *not completed: questionnaire was sent to participant, but participant did not complete the questionnaire. The participant was not lost-to-follow-up. ^ǂ^e.g. takes too much time or effort, too complicated, not able to participate due to other illness. ^×^The questionnaire was not sent to a participant because the study stopped before the participant reached this follow-up point).

A total of 55 general practices participated in the study, with 41 participating in the intervention group (i.e. 31 using the ABCC-tool via the chain care information system, 10 using the GP information system) and 14 participating in the control group. Healthcare providers (i.e. general practitioners, general practice nurses, and nurse practitioners) in the intervention group and the control group included 176 and 61 patients, respectively (Figure 2). The baseline characteristics of the 235 patients are shown in Table 1 and Supplementary Table 1. At baseline, 35 patients were diagnosed with COPD, 28 patients were diagnosed with asthma, 197 patients were diagnosed with type 2 diabetes, and 30 patients were diagnosed with heart failure. In total 188 patients were diagnosed with one of the four chronic conditions and 47 patients were diagnosed with a combination of the four chronic conditions (see Supplementary Table 2 for the combination of those chronic conditions).

**Table 1.** Baseline characteristics.

	Intervention group (*n* = 176)	Control group (*n* = 59)	*p* value
Age, years, mean (SD)	63.2 (9.3)	64.0 (10.4)	0.604^1^
Sex, male, n (%)	120 (68.2)	41 (69.5)	0.851^2^
Smoking status, n (%)			
Never smoked	57 (32.4)	20 (33.9)	0.787^2^
Ex-smoker	95 (54.0)	33 (55.9)	
Current smoker	24 (13.6)	6 (10.2)	
BMI, kg/m^2^, mean (SD)	29.7 (5.9)	29.2 (4.5) *Missing n* = 1	0.521^1^
Diagnosed with COPD	28 (15.9)	7 (11.9)	0.450^2^
Modified medical research			
Council Dyspnoea questionnaire			
0	0 (0.0)	0 (0.0)	0.659^3^
1	13 (46.4)	5 (71.4)	
2	11 (39.3)	2 (28.6)	
3	3 (10.7)	0 (0.0)	
4	1 (3.6)	0 (0.0)	
Diagnosed with asthma	13 (7.4)	15 (25.4)	<0.001^2^
Asthma control test; mean (SD)*	18.7 (4.2)	18.6 (5.2)	0.977^1^
Asthma control test			
Not well controlled	4 (30.8)	10 (66.7)	0.128^4^
Well controlled	9 (69.2)	5 (33.3)	
Diagnosed with type 2 diabetes	152 (86.4)	45 (76.3)	0.068^2^
Complications due to type 2 diabetes			
Nephropathy	4 (2.7)	4 (8.9)	0.085^4^
Neuropathy	14 (9.2)	5 (11.1)	0.774^4^
Eye complications	8 (5.3)	1 (2.2)	0.687^4^
Sexual complications	30 (19.7)	9 (20.0)	0.969^2^
Amputation	0 (0.0)	0 (0.0)	NA^5^
Diabetic foot	2 (1.3)	2 (4.4)	0.225^4^
Cardiovascular disease	33 (21.7)	11 (24.4)	0.699^2^
*Missing*	2	–	NA
Diagnosed with heart failure	20 (11.4)	10 (16.9)	0.266^2^
NYHA functional classification			
NYHA 1	8 (42.1)	3 (30.0)	0.696^3^
NYHA 2	6 (31.6)	5 (50.0)	
NYHA 3	5 (26.3)	2 (20.0)	
NYHA 4	0 (0.0)	0 (0.0)	
*Missing*	1	–	

* score ranges from 5 (poor control of asthma) to 25 (complete control of asthma); NYHA = New York Heart Association; ^a^Independent sample T-test; ^b^Chi square test; ^c^Fishers-Freeman-Halton exact test (when one or multiple cells had expected frequencies of <5 in a m x n table); ^d^Fisher’s Exact Test (when one or multiple cells had expected frequencies of <5 in a 2 x 2 table); ^e^Not applicable.

### Intervention compliance

In the intervention group, 130 out of 176 patients (73.9%) used the ABCC-tool at least once during the study. The ABCC-scale was completed 258 times: 221 times by people with type 2 diabetes (85.7%), 14 times by people with COPD (5.4%), 9 times by people with asthma (3.5%), 11 times by people with type 2 diabetes and asthma (4.3%), and 3 times by people with type 2 diabetes and COPD (1.2%). The heart failure module was not used. Among those who used the ABCC-tool, use ranged from one to six times (median: 2) in 18 months. Out of the 258 times the ABCC-tool was used, a goal was formulated 56 times (21.7%). All patients were included in the analysis, regardless of whether they used the ABCC-tool.

### Primary outcome

#### Perceived quality of care after 18 months for the total group

**Figure 3.**
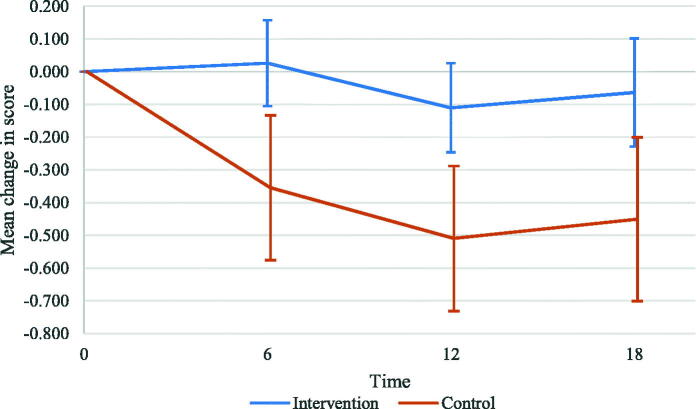
Mean change in PACIC total scores at 6, 12, and 18-month follow-up compared with baseline for the total group. The whiskers show the 95% confidence intervals.

We observed a significant effect of the ABCC-tool on the PACIC total score for the total group after 18 months (0.388 points; 95%CI: 0.089–0.687; *p* = 0.011). The score in the intervention group remained approximately stable over time, while the score in the control group decreased (Table 2; Figure 3; and output in Supplementary Tables). Effects of potential confounders on the total score of the PACIC are reported in Supplementary Table 3.

**Table 2.** Effect of the ABCC-tool on the total score and subdomains of the **PACIC** at T6, T12, and T18 for the **total group**; observed outcomes and intervention effects as established with mixed linear regression and corrected for potential confounders*.

				95%CI		
	Score in intervention group, mean (SD); n	Score in control group, mean (SD); n	B**	Lower	Upper	*p* value
Total score						
Baseline	3.06 (0.82); 174	3.17 (0.81); 59	–	–	–	–
6 months	3.10 (0.79); 150	2.83 (0.85); 54	0.381	0.125	0.638	**0.004**
12 months	2.98 (0.90); 132	2.68 (0.84); 50	0.400	0.141	0.659	**0.002**
18 months	3.05 (0.89); 91	2.73 (0.97); 44	0.388	0.089	0.687	**0.011**
Patient activation						
Baseline	3.69 (1.13); 175	3.95 (1.10); 59	–	–	–	–
6 months	3.63 (1.20); 150	3.36 (1.17); 54	0.512	0.097	0.927	**0.016**
12 months	3.53 (1.16); 132	3.22 (1.12); 50	0.548	0.146	0.949	**0.007**
18 months	3.53 (1.22); 91	3.16 (1.16); 44	0.734	0.337	1.131	**<0.01**
Delivery system design/decision support						
Baseline	3.54 (0.82); 175	3.69 (0.84); 59	–	–	–	–
6 months	3.56 (0.76); 150	3.31 (0.81); 54	0.396	0.133	0.659	**0.003**
12 months	3.44 (0.86); 131	3.12 (0.91); 50	0.411	0.145	0.677	**0.002**
18 months	3.44 (0.81); 91	3.17 (0.86); 44	0.350	0.031	0.670	**0.023**
Goal setting/tailoring						
Baseline	2.84 (0.90); 174	2.97 (0.97); 59	–	–	–	–
6 months	2.95 (0.86); 150	2.63 (0.94); 54	0.468	0.189	0.746	**<0.001**
12 months	2.82 (1.00); 131	2.46 (0.87); 50	0.514	0.226	0.801	**<0.001**
18 months	2.80 (0.93); 91	2.59 (1.10); 44	0.305	−0.037	0.647	0.081
Problem solving/contextual						
Baseline	3.33 (1.09); 174	3.32 (1.16); 59	–	–	–	–
6 months	3.51 (1.10); 150	3.09 (1.16); 54	0.393	0.020	0.766	**0.039**
12 months	3.29 (1.17); 132	2.86 (1.13); 50	0.426	0.056	0.797	**0.024**
18 months	3.33 (1.17); 91	2.90 (1.25); 44	0.412	0.017	0.807	**0.041**
Follow-up/coordination						
Baseline	2.37 (0.91); 174	2.48 (1.00); 59	–	–	–	–
6 months	2.33 (0.87); 150	2.21 (0.89); 54	0.206	−0.075	0.486	0.151
12 months	2.31 (0.92); 131	2.16 (0.88); 50	0.217	−0.072	0.506	0.141
18 months	2.54 (0.95); 91	2.23 (1.00); 44	0.281	−0.055	0.616	0.101

Scale ranges from 1 to 5. *Adjusted for all potential confounders; **B = mixed linear regression weight for treatment, indicating the estimated difference between intervention and control at 6, 12 or 18 months, corrected for the outcome at baseline and potential confounders. *B* > 0 indicates a higher score in the intervention group. ABCC-tool = Assessment of Burden of Chronic Conditions tool; PACIC = Patient Assessment of Chronic Illness Care.

### Secondary outcomes

#### Perceived quality of care

We found significant effects of the ABCC-tool on the PACIC subdomains patient activation, delivery system design/decision support, and problem-solving/contextual after 18 months (Table 2). After 6 and 12 months, we found significant effects of the ABCC-tool on the PACIC total score (Table 2 and Figure 3). The estimated mean change in the PACIC total score was 0.381 points (95%CI: 0.125–0.638; *p* = 0.004) and 0.400 points (95%CI: 0.141–0.659; *p* = 0.002) at 6 and 12 months, respectively. The analysis of the PACIC total score for type 2 diabetes showed significant intervention effects after 6 and 12 months, but not after 18 months (Supplementary Table 4). All significant effects were in favour of the intervention group.

### Generic health-related quality of life

No significant intervention effect on the EQ-5D-5L was found. At 18 months, the estimated mean change for the total group was 0.032 points (95%CI: −0.005–0.069; *p* = 0.091) and 0.691 points (95%CI: −4.031–5.414; *p* = 0.774) for the EQ-5D-5L index score and VAS, respectively (Supplementary Tables 5 and 6).

### Patients’ activation

We observed a significant effect of the ABCC-tool on the PAM after 18 months for the total group (5.768 points; 95%CI: 0.776–10.760; *p* = 0.024), but not for type 2 diabetes (Supplementary Tables 7 and 8 and Supplementary Figure 1). No significant intervention effect was found after 6 and 12 months.

### Capability well-being

No significant effect of the ABCC-tool on the ICECAP-A was found after 6, 12, and 18 months for the total group and for type 2 diabetes (Supplementary Tables 9 and 10). The estimated mean change for the total group was 0.024 points (95%CI: −0.009–0.056; *p* = 0.155) at 18 months.

### Sensitivity analyses

Results of the sensitivity analysis are provided in Supplementary Material 7 and Supplementary Tables 11–22. Selection of potential confounders, a per-protocol analysis, and inclusion of physical consultations in the model did not result in different conclusions regarding the primary outcome.

## Discussion

### Main findings

In this pragmatic clustered quasi-experimental study, we observed a significant effect of the ABCC-tool on perceived quality of care after 6, 12, and 18 months. Use of the ABCC-tool also had a significant effect on patients’ activation after 18 months. All significant effects were in favour of the intervention group. It should be noted that the score in the intervention group remained approximately stable over time, while the score in the control group decreased, indicating a worse perceived quality of care. Inferences regarding patients’ activation should be made with caution, given that this was our secondary outcome. However, the score in patients’ activation as measured by the PAM decreased by more than four points in the control group, indicating a minimal clinically important difference [17]. This might underline our finding that the ABCC-tool positively affects the patients’ activation. The main analysis found no significant effects on generic health status and capability well-being.

### Interpretation of the study

As the ABCC-tool can be described as a complex intervention, it is important to understand how it contributes to change [18]. Supplementary Figure 2 shows how the ABCC-tool is expected to lead to its outcomes and impacts, including the necessary conditions. Because the effects of complex interventions often depend strongly on the context [18], the effects of the ABCC-tool should be interpreted within its context. For example, the different characteristics of the general practices (e.g. the progressiveness of the general practice or the age of healthcare providers) may have influenced the willingness to participate and the way the intervention was used. The study results may also have been influenced by the increased pressure on chronic care in the Netherlands, due to among others the COVID-19 pandemic, increased demand for care, and increased complexity of care. In addition, the results should be interpreted in light of the COVID-19 pandemic, which most likely played a significant role in the dynamics between the intervention and its outcomes. For example, policy decisions related to disruption of healthcare for people with chronic conditions may have influenced outcomes. Furthermore, the recruitment of general practices may have been impacted by the COVID-19 pandemic, as we noticed it was easier to recruit intervention practices than control practices during the COVID-19 pandemic (Supplementary Figures 3 and 4). The COVID-19 pandemic may also have influenced which patients were recruited for the study. To better understand why and how outcomes and impacts were (not) achieved, a context and process evaluation have been conducted among healthcare providers concomitantly to this study [19]. The results will be published elsewhere.

### Comparison with existing literature

In line with our study, the study on the effectiveness of the ABC-tool – the predecessor of the ABCC-tool – showed significant intervention effects on the PACIC total score after 18 months [8]. We did not find a significant impact of the ABCC-tool on generic quality of life. This is in line with other studies of interventions aimed at person-centred care [20, 21]. Possible explanations for these findings are that the effects are more disease-specific for which the (generic) EQ-5D-5L is not suitable. Furthermore, longer periods might be needed before benefits become apparent or effects can only be detected in patients who were actually treated with the ABCC-tool (as we did find significant effects in the per protocol analysis). We found a significant intervention effect on patients’ activation after 18 months, but not after 6 and 12 months. A systematic review and meta-analysis on patient activation interventions identified no significant intervention effect on the PAM after 6 months [22], which is in line with our findings. This might indicate that a longer follow-up time is needed to measure improvements in patients’ activation. To our knowledge, no study on person-centred care, shared decision-making, or self-management for chronic conditions has been conducted that included the ICECAP-A as an outcome measure.

### Strengths and limitations

This study has several strengths. The study has a pragmatic approach in real-life routine practice with broad inclusion criteria. It was conducted in line with recommended standards.

The study also has several limitations. First, the study was conducted during the COVID-19 pandemic. We tried to correct for COVID-19 in several ways (Supplementary Material 8). Nevertheless, we cannot ensure our results are valid in a post-COVID-19 situation.

Second, due to the pragmatic design, using the ABCC-tool was not actively encouraged during the study. Low intervention use probably diluted the intervention effect.

Third, for pragmatic reasons, we did not apply targeted implementation strategies. We expect that with more effective strategies for implementing the ABCC-tool, the effect might have been larger.

Fourth, partly due to the COVID-19 pandemic, recruiting practices and participants was challenging. This resulted in a lower sample size than the required one, which diminished its power. Furthermore, the number of participants in the intervention and control groups were unequally divided (i.e. 176 patients in the intervention group compared to 59 patients in the control group) further decreasing the power. The disbalance between the intervention and control groups is likely due to the COVID-19 pandemic. Supplementary Figures 3 and 4 show that the recruitment in both groups changed with the start of the pandemic. The observed standardised effect size for the primary outcome was lower than the one we used for sample size calculation (0.35 versus 0.51), yielding a lower power than prespecified. On the contrary, the ICC for the primary outcome was 0.033, while an ICC of 0.050 was used in the sample size calculation. The study population consisted largely of patients diagnosed with type 2 diabetes. Therefore, there is less evidence for people with COPD and asthma. Furthermore, since the heart failure module was not used, we are not able to conclude the effect of the heart failure module. In the Netherlands, patients with heart failure are less monitored by practice nurses, leading to the non-use of this module. However, patients with heart failure and other conditions did use the ABCC-tool with the module for the other conditions.

Fifth, the dropout of participants in the intervention group (17%) and in the control group (14.8%) may have resulted in attrition bias.

Sixth, no minimal clinically important difference has been defined for the PACIC [23]. Therefore, it is unclear whether the intervention effects are clinically relevant.

Seventh, the lack of randomisation might have caused an imbalance in baseline values and potential confounders between both groups. However, the differences between the groups at baseline were minimal, all hypothesised potential confounders were included in the analysis, and we used a hierarchical model. Different GPs use different information systems which might introduce an allocation bias. However, as the ABCC-tool was used via the chain care information system in 31 of the 41 cases, we expect this bias to be negligible, as the chain care information system is not chosen by the individual healthcare provider but by the care group.

Eighth, we did not correct for multiple testing, thereby increasing the risk of false positive outcomes.

Last, both healthcare providers and researchers were not blinded. The first could have resulted in selection bias.

### Recommendations for further research

We recommend replication of the trial to endorse the results, especially after the COVID-19 pandemic. Furthermore, we recommend the development of modules for more common chronic conditions that can be added to the ABCC-tool. Currently, the ABCC-tool is extended to be suited for osteoarthritis and cardiovascular risks. We are planning a study on the cost-effectiveness of the ABCC-tool using data on medical consumption and productivity costs collected during this trial. Interviews will be conducted with patients who participated in this study to evaluate their experiences in using the ABCC-tool. The ABCC-tool can be translated and validated, and if necessary, culturally adapted, to be suitable for use in other countries as well. In South-Tirol a study will be conducted using the ABCC-tool to facilitate personalised care [24].

## Conclusion

The ABCC-tool may provide high-quality, accessible, and affordable care to increasing numbers of people with one or multiple chronic condition(s). It aims to enable a person-centred approach by providing insight into the burden of disease including social, emotional, and physical aspects. Furthermore, it offers adequate support for shared decision-making and self-management through visualisation of disease burden. Lastly, it combines multiple chronic conditions in one tool, providing patients with one individual care plan that addresses multiple chronic conditions and avoids overlap. Our results showed that using the ABCC-tool during consultations in general practices for people with chronic conditions has a positive effect on the perceived quality of care and on patients’ activation. It might thus be of added value for care for people with chronic conditions.

Box 1 The Assessment of Burden of Chronic Conditions (ABCC)-tool

The ABCC-tool integrates a PROM about the experienced burden of chronic conditions with lifestyle assessment, together with a visualisation of the scores using balloons (Figure 1) and generic treatment advice. The patient completes the questionnaire before consultation with the healthcare provider. The results of this questionnaire are visualised into balloons, with the colour and height of the balloon indicating the score on a domain. This allows for easy identification of areas that need specific attention and for engagement of the patients in their treatment. The patient and healthcare provider can discuss the domains and click on a balloon. Treatment advice based on current clinical guidelines appears, that can help to compose a personalised care plan together, including individualised goals and action plans, based on the needs and wishes of a patient and the treatment options available. If the patient follows the care plan and achieves its goals, the balloon for that domain will move to a higher position in the visualisation the next time the ABCC-tool is used. Previous results are displayed using grey balloons, which allows to easily detect change from the previous measurement. The tool has shown to be a valid and reliable instrument for evaluating the experienced burden of disease for people with COPD, asthma, type 2 diabetes, and heart failure.

## Supplementary Material

Supplemental Material

## Data Availability

The datasets have been deposited in the DataHub Maastricht Repository, under accession number 21.12109/P000000332C000000003. Parties interested in the ABCC-tool, including its specifications and requirements for incorporation in IT-systems, can contact the corresponding author of this article via email. The ABCC-tool is free of charge, on the condition of correct acknowledgement and no modifications. The availability of the ABCC-tool to the GP practice will depend on the IT-provider.
