# Supplementary material for: Effectiveness of the Assessment of Burden of Chronic Conditions (ABCC)-tool in patients with asthma, COPD, type 2 diabetes mellitus, and heart failure: A pragmatic clustered quasi-experimental study in the Netherlands
Source: Eur J Gen Pract. 2024 May 13;30(1):2343364. doi: 10.1080/13814788.2024.2343364 (PMC11104697; doi:10.1080/13814788.2024.2343364)
Supplement: Supplemental Material [file IGEN_A_2343364_SM0531.docx]

**Supplementary Material**

**Content Supplementary Material**

1. Deviations from the study protocol Page 2
2. Design choices – PRECIS-2 tool Page 4
3. Description of the intervention – TIDieR checklist Page 5
4. Measurements Page 6
5. Syntax primary outcome Page 6
6. Methods sensitivity analyses Page 6
7. Results sensitivity analyses Page 6
8. Correcting for COVID-19 Page 7

**Content Supplementary Tables**

*Baseline characteristics*

1. Baseline characteristics regarding potential confounders Page 8
2. Baseline characteristics regarding chronic conditions Page 8
3. Effects of potential confounders on the total score of the PACIC Page 9

*Secondary outcomes*

1. Effect of ABCC-tool on the PACIC for type 2 diabetes Page 10
2. Effect of ABCC-tool on the EQ-5D-5L for the total group Page 10
3. Effect of ABCC-tool on the EQ-5D-5L for type 2 diabetes Page 11
4. Effect of ABCC-tool on the PAM for the total group Page 11
5. Effect of ABCC-tool on the PAM for type 2 diabetes Page 11
6. Effect of ABCC-tool on the ICECAP-A for the total group Page 12
7. Effect of ABCC-tool on the ICECAP-A for type 2 diabetes Page 12

*Sensitivity analyses*

1. Effect of ABCC-tool on the PACIC; selection potential confounders Page 13
2. Effect of ABCC-tool on the EQ-5D-5L; selection potential confounders Page 13
3. Effect of ABCC-tool on the PAM; selection potential confounders Page 14
4. Effect of ABCC-tool on the ICECAP-A; selection potential confounders Page 14
5. Effect of ABCC-tool on the PACIC; per protocol Page 15
6. Effect of ABCC-tool on the EQ-5D-5L; per protocol Page 15
7. Effect of ABCC-tool on the PAM; per protocol Page 16
8. Effect of ABCC-tool on the ICECAP-A; per protocol Page 16
9. Effect of ABCC-tool on the PACIC; including physical consultations Page 17
10. Effect of ABCC-tool on the EQ-5D-5L; including physical consultations Page 17
11. Effect of ABCC-tool on the PAM; including physical consultations Page 18
12. Effect of ABCC-tool on the ICECAP-A; including physical consultations Page 18

**Content Supplementary Figures**

1. Mean change in PAM scores Page 19
2. Logic model of the ABCC-tool Page 19
3. Completed baseline questionnaires per month Page 20
4. Number of general practices included in the study per month Page 20

**References**  Page 21

**Supplementary Material 1: Deviations from the study protocol**

| **Protocol** | **Study** | **Reason** |
| --- | --- | --- |
| The study will have a follow-up period of 18 months. | Not all patients completed the follow-up time of 18 months. | Although not every patient had completed the follow-up time by November 2022, we had to stop collecting data for feasibility reasons. |
| A tariff value for the overall state for the ICECAP-A will be determined based on values in the UK. | Dutch tariff values were used for the overall state for the ICECAP-A. | Dutch tariff values were developed during the course of our study. |
| Secondary outcomes include:  1. Change in perceived quality of care, as measured by the PACIC, compared with usual care after 18 months for each condition separately.  2. Change in perceived quality of care, as measured by the PACIC, compared with usual care after 6 and 12 months for the total group and for each condition separately | Secondary outcomes included:  1. Change in the PACIC subdomains after 18 months for the total group  2. Change in the PACIC (total score and subdomains) after 18 months for chronic conditions separately  3. Change in the PACIC (total score and subdomains) after 6 and 12 months for the total group and chronic conditions separately. | The total score and subdomains of the PACIC have been reported separately. |
| Multiple imputation will be used to correct for missing data. | Multiple imputation was used to correct for missing data at baseline, but not for outcomes. | The imputation model would become too large. |
| The effects of the ABCC tool for patients with COPD, asthma, type 2 diabetes and heart failure will be analysed separately. | The effects of the ABCC tool were only separately analysed for type 2 diabetes. | Due to the small number of patients with COPD, asthma, and heart failure (35, 28, and 30 patients at baseline respectively), we decided to not conduct separate analyses for each condition. |
| Each potential confounder will be separately added to the linear mixed model, and will be considered as a confounder if significantly associated with the outcome (p≤0.05). | All potential confounders were included in our main analysis. | We expected that the analysis could not be conducted with all potential confounders included in the model. However, our sample size was large enough to include all potential confounders in the model. |
| Some confounders differ from the confounders stated in the protocol. Firstly, instead of using multimorbidity (yes/no), we decided to include the following confounders: asthma (yes/no); COPD (yes/no); type 2 diabetes (yes/no); heart failure (yes/no); and other disease (yes/no). These five confounders would have been merged into multimorbidity (yes/no). However, we decided to include the five confounders separately, because the various chronic conditions were not equally divided among the intervention and control group. If care for one of the chronic conditions was temporally cancelled during COVID-19, this disruption – which could lead to a lower perceived quality of care - might not be equal for the intervention and control group. Secondly, we included diagnosed COVID-19 (yes/no) as potential confounders, as the COVID-19 pandemic started soon after the start of the study and it might influence both the use of the intervention as well as perceived quality of care. | | |
| As a sensitivity analysis, an inverse probability of treatment weighting using propensity scores will be applied to account for multiple potential confounders. | We conducted several sensitivity analyses. First, we used a backward elimination method to select potential confounders. Second, a per protocol analysis was conducted, in which all patients in the control group and only patients in the intervention group who had received the intervention for at least one time were included. Last, we included the number of physical consultations in our model. | 1. In our final model, all potential confounders mentioned in the protocol were included. Therefore, we decided to select potential confounders using a backward elimination method. 2. As 73.9% of the study participants used the intervention, we assessed the effect in patients who had received the intervention for at least one time. 3. See column below about the COVID-19 pandemic. |
| The study team will be blinded to the treatment arms in the dataset during data cleaning, handling of missing data, statistical analysis and drawing of conclusions. | The researchers were not blinded. | Blinding of researchers was not possible, as the researchers did already know of the number of participants in each group. |
| The study was conducted during the COVID-19 pandemic. We tried to correct for COVID-19 in three ways. First, we included a diagnosis of COVID-19 as potential confounder in our model. Second, we included COPD, asthma, type 2 diabetes, and heart failure as potential confounders in our model. The reason is that these chronic conditions were not equally distributed between the intervention and control group. If care for one of the chronic conditions was temporally cancelled during COVID-19, this disruption – which could lead to a lower perceived quality of care - might not be equal for the intervention and control group. Lastly, we included the number of physical consultations as a confounder in the sensitivity analysis for the same reason as mentioned above, but with the possibility to control for changes in care over time. | | |

**Supplementary Material 2: Design choices – PRECIS-2 tool**

Based on the PRagmatic Explanatory Continuum Indicator Summary-2 (PRECIS-2) tool


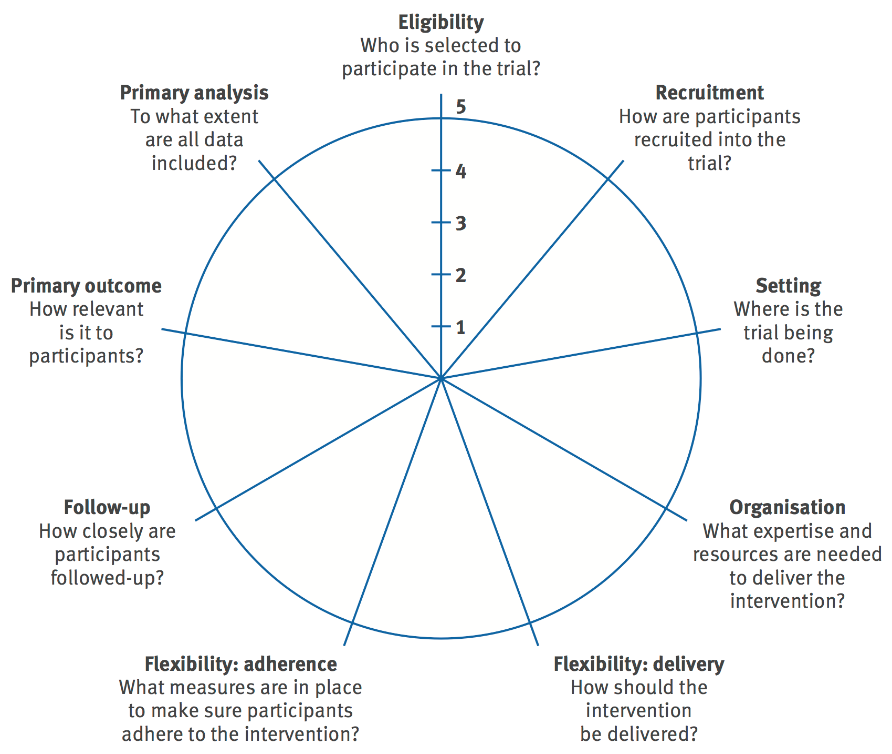


| **Domain** | **Score*** | **Rationale** |
| --- | --- | --- |
| Eligibility | 4 | Only a few eligibility criteria have been set. Healthcare providers were recruited without specific criteria or prerequisites but were excluded from the control group if they had already used the ABC-tool (the predecessor of the ABCC-tool). Concerning the intervention group, healthcare providers were allowed to have used the ABC-tool. Patients were eligible if they have the diagnosis of COPD, asthma, type 2 diabetes and/or heart failure, were aged 18 or older, and could understand and read the Dutch language. To ensure that participants were stable at baseline, patients with asthma or COPD were not eligible if they had used prednisone due to an exacerbation within the 6-week period prior to the commencement of the study, and patients with type 2 diabetes or heart failure were not eligible if they were hospitalised within the 6-week period prior to the commencement of the study. Patients who had already used the ABC tool were also be excluded |
| Recruitment | 4 | Patients were recruited by healthcare providers. Healthcare providers were asked to invite patients to participate in the research prior to their consultation (a bit of extra effort compared to the usual care setting). If a patient wanted to participate and was eligible, the healthcare provider registered the patient on a website. |
| Setting | 5 | The setting is identical to usual care. The intervention group used the ABCC-tool during regular consultations. |
| Organisation | 4 | There was no increase in resources (e.g. money or staff) nor was expertise required. Healthcare providers in the intervention group received a short instructional film about the ABCC tool before the start of the study as well as a poster that shortly displayed the steps of the ABCC tool. This might differ from the usual care setting, although we expect that healthcare providers in a usual care setting also receive a training about the use of the ABCC-tool. |
| Flexibility (delivery) | 5 | The flexibility of in how the intervention is delivered is identical to usual care. There were no strict protocols, monitoring, or measures to improve compliance. |
| Flexibility  (adherence) | 5 | The flexibility in how participants must adhere to the intervention is identical to usual care. The study included no more than usual encouragement to adhere to the intervention. |
| Follow-up | 5 | The intervention group used the ABCC-tool during regular consultations. The intervention did not result in more frequent or longer visits. |
| Primary outcome | 5 | The primary outcome is perceived quality of care, which is of importance to participants. |
| Primary analysis | 5 | Data were analysed according to the intention-to-treat principle, including all available data. |

*Scored from 1 (very explanatory) to 5 (very pragmatic)

**Supplementary Material 3: Description of the intervention – TIDieR checklist**

Based on the Template for Intervention Description and Replication (TIDieR) checklist

Brief name: The Assessment of Burden of Chronic Conditions (ABCC) tool

Why: The ABCC-tool may improve insight in burden of disease, facilitate shared decision making between the healthcare provider and patient, and increase patients’ activation and self-management by composing a personalised care plan. This might lead to care that is patient-centered, proactive, planned and includes collaborative goal setting, problem-solving and follow-up support. Or, in other words, to improved perceived quality of care. Furthermore, by using the ABCC-tool, healthcare providers can help patients to become more self-confident and skilled to manage their own disease. This might, in the long-term, lead to healthier lifestyles and improved quality of life and patients’ capabilities.

What (material): The intervention group used the ABCC-tool. Box 1 provides a more detailed description of how the ABCC-tool works. The ABCC-tool was implemented in the information system of the healthcare providers. Healthcare providers in the intervention group received a short instructional film about the ABCC-tool before the start of the study as well as a poster that shortly displays the steps of the ABCC-tool. Interested parties can contact the corresponding author of this article via email to receive the specifications of the ABCC-tool, the instructional film, or the poster.

What (procedures): Healthcare providers were trained to use the ABCC-tool using the short explainer film and poster. Patients willing to use the ABCC-tool were recruited by healthcare providers. The ABCC-tool was used in consultations between the healthcare providers and patients.

Who provided: The intervention was provided by healthcare providers, mainly by the general practice nurse. No specific expertise or background were required, nor was specific training needed. Healthcare providers in the intervention group received a short instructional film about the ABCC-tool before the start of the study as well as a poster that shortly displays the steps of the ABCC-tool.

How: In the Netherlands, there are different systems for managing electronic medical records. The ABCC-tool was implemented in two information systems in the Netherlands, entitled ‘Sananet’ and ‘NHGDoc’. Patients who used Sananet could complete the ABCC-questionnaire before the consultation between the healthcare provider and patient using an online platform. Patients who used NHGDoc completed a paper version of the ABCC-questionnaire in the waiting room of the general practice. For both groups, the results were visualised and discussed, and treatment plans were formulated, during the consultation at the general practice. This was face to face and individually.

Where: The study was conducted in general practices in the Netherlands. The intervention group either used Sananet or NHGDoc as information system (see ‘how’).

When and how much: The intervention group used the ABCC-tool during regular consultations, which takes approximately 20 minutes. The frequency of visits depends on several factors, which are described in the guidelines of the Dutch College of General Practitioners. In general, patients with type 2 diabetes or heart failure visit their healthcare provider four times a year; patients with COPD with mild burden of disease or patients with well-controlled asthma visit their healthcare provider once a year. Therefore, it was expected that patients with COPD or asthma use the ABCC tool once or twice during the study, and patients with type 2 diabetes or heart failure will use the ABCC tool about six times during the study.

Tailoring: The essence of the intervention is that it can be personalised, although the core elements (measuring burden of disease, visualising burden of disease, discussing treatment options, formulating goals and action plans, and monitoring) are not adapted. However, patients and healthcare providers together decide on the domain they want to discuss and the goal they want to work on.

Modifications: There were no unforeseen modifications to the intervention.

How well (planned): In the intervention group, 130 out of 176 patients (73.9%) used the ABCC-tool at least one time during the study.

How well (actual): Fidelity of the intervention will be described in another scientific study.

**Supplementary Material 4: Measurements**

The PACIC assesses the extent to which patients with chronic conditions receive care that aligns with the Chronic Care Model (1, 2). It consists of 20 items across five subdomains: patient activation (3 items), delivery system design/decision support (3 items), goal setting/tailoring (5 items), problem-solving/contextual (4 items), and follow-up/coordination (5 items). Answers range from 1 (almost never) to 5 (almost always), with higher scores indicating more frequent presence of the aspect of structured chronic care.

The EQ-5D-5L is a measure of generic health status, using five dimensions: mobility, self-care, usual activities, pain/discomfort, and anxiety/depression (3, 4). Answers range from 1 (no problems) to 5 (extreme problems). These answers can be converted into a single index value using a Dutch value set. The EQ-5D-5L also includes a Visual Analogue Scale (VAS) to measure self-rated health, with endpoints labelled ‘the best health you can imagine’ to ‘the worst health you can imagine’.

The PAM measures people’s knowledge, skills and confidence (referred to as ‘patient activation’) in managing their own wellbeing (5, 6). Answers include four categories of agreement, ranging from ‘disagree strongly’ to ‘agree strongly’. The activation score ranges from 0 (lowest possible activation) to 100 (highest possible activation). It was decided to include both the PACIC subdomain patient activation as well as the PAM, as the PACIC has shown only moderate correlations with measures of patient activation (1). We did not assess the correlation between the PACIC and the PAM, as this was not the aim of our study.

The ICECAP-A assesses five capabilities that are important to one’s quality of life, including stability, attachment, autonomy, achievement, and enjoyment (7). Answers range from 1 (no capability) to 4 (full capability). The study protocol stated that tariff values would be based on those from the United Kingdom (8). However, Dutch tariff values were developed during the course of our study and therefore it was decided to use the Dutch tariff values (9).

**Supplementary Material 5: Syntax primary outcome**


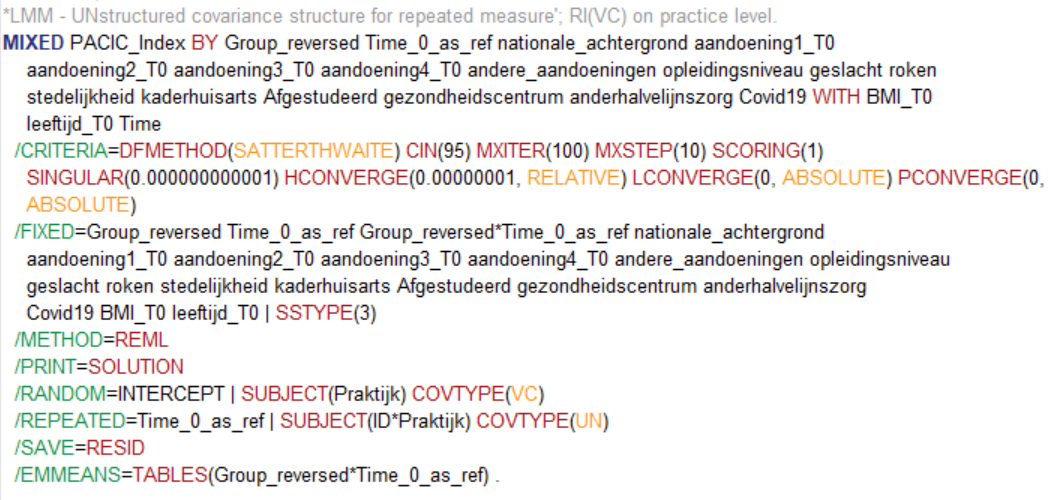


**Supplementary Material 6: Methods sensitivity analyses**

We conducted several sensitivity analyses. First, we used a backward elimination method to select potential confounders. Second, a per protocol analysis was conducted, in which all patients in the control group and only patients in the intervention group who had received the intervention for at least one time were included. Last, we included the number of physical consultations in our model.

**Supplementary Material 7: Results sensitivity analyses**

The selection of potential confounders did not result in different conclusions, except for the ICECAP-A, in which the intervention effect became significant after 12 months (Supplementary Tables 10-13). The per protocol analysis did not show any different intervention effect with four exceptions: a significant effect of the ABCC-tool on the EQ-5D-5L index after 18 months and on the ICECAP-A after 12 and 18 months, and a non-significant effect on the PAM after 18 months and on the PACIC subdomain problem solving after 6 and 18 months (Supplementary Tables 14-17). Including physical consultations in the model, besides all other potential confounders, did not result in different conclusions, except for the PACIC subdomain problem solving after 6 months (non-significant) and the EQ-5D-index after 18 months (significant) (Supplementary Tables 18-21).

**Supplementary Material 8: Correcting for COVID-19**

The study was conducted during the COVID-19 pandemic. We tried to correct for COVID-19 in three ways. First, we included a diagnosis of COVID-19 as potential confounder in our model. Second, we included COPD, asthma, type 2 diabetes, and heart failure as potential confounders in our model. The reason is that these chronic conditions were not equally distributed between the intervention and control group. If care for one of the chronic conditions was temporally cancelled during COVID-19, this disruption – which could lead to a lower perceived quality of care - might not be equal for the intervention and control group. Lastly, we included the number of physical consultations as a confounder in the sensitivity analysis for the same reason as mentioned above, but with the possibility to control for changes in care over time.

***Baseline characteristics***

**Supplementary Table 1: Baseline characteristics regarding potential confounders**

|  | **Intervention group (n=176)** | **Control group**  **(n=59)** | **p-value** |
| --- | --- | --- | --- |
| National background, n (%)  Dutch  1^st^ generation migrant  2^nd^ generation migrant  Missing | 152 (86.4)  8 (4.5)  16 (9.1)  - | 53 (91.4)  1 (1.7)  4 (6.9)  1 | 0.676 ^1^ |
| Educational level, n (%)  Low  Medium  High  Missing | 53 (30.3)  61 (34.9)  61 (34.9)  1 | 27 (45.8)  17 (28.8)  15 (25.4)  - | 0.091 ^2^ |
| Other diseases | 76 (43.2) | 23 (39.0) | 0.572 ^2^ |
| Location of general practice  Urban  Rural | 120 (68.2)  56 (31.8) | 35 (59.3)  24 (40.7) | 0.214 ^2^ |
| General practitioner with specialisation in COPD, asthma, type 2 diabetes, or heart failure  COPD/asthma  Heart and vascular disease  Both | 22 (12.5)  5 (16.1)  21 (67.7)  5 (16.1) | 0  0  0  0 | 0.002 ^3^  N/A  N/A  N/A |
| Year of graduation managing general practitioner  <10 years ago  >10 years ago  Missing | 22 (12.6)  153 (87.4)  1 | 3 (5.1)  56 (94.9)  - | 0.144 ^3^ |
| General practice in a health centre  Yes  No | 112 (64.0)  63 (36.0) | 33 (55.9)  26 (44.1) | 0.270 ^2^ |
| Possibility of consulting a specialist within primary care  Yes  No | 167 (94.9)  9 (5.1) | 50 (84.7)  9 (15.3) | 0.020 ^3^ |

*^1^Fishers-Freeman-Halton exact test (when one or multiple cells had expected frequencies of <5 in a m x n table); ^2^Chi square test; ^3^Fisher’s Exact Test (when one or multiple cells had expected frequencies of <5 in a 2x2 table)*

**Supplementary Table 2: Baseline characteristics regarding chronic conditions**

|  | **Intervention group (n=176)** | **Control group**  **(n=59)** | **p-value** |
| --- | --- | --- | --- |
| Diagnosed with, n (%)  COPD  Type 2 diabetes  Heart failure  Asthma  COPD and asthma  COPD and type 2 diabetes  Asthma and type 2 diabetes  Type 2 diabetes and heart failure  Asthma, type 2 diabetes, and heart failure  COPD, asthma, and type 2 diabetes  COPD, asthma, and heart failure  COPD, type 2 diabetes, and heart failure  COPD, asthma, type 2 diabetes, and heart failure | 17 (9.7)  119 (67.6)  2 (1.1)  5 (2.8)  0 (0)  8 (4.5)  6 (3.4)  16 (9.1)  0 (0)  1 (0.6)  0 (0)  1 (0.6)  1 (0.6) | 2 (3.4)  34 (57.6)  1 (1.7)  8 (13.6)  2 (3.4)  1 (1.7)  2 (3.4)  6 (10.2)  1 (1.7)  0 (0)  1 (1.7)  0 (0)  1 (1.7) | 0.170^1^  0.164^2^  1.000^1^  **0.005**^1^  0.062^1^  0.456^1^  1.000^1^  0.806^2^  0.251^1^  1.000^1^  0.251^1^  1.000^1^  0.440^1^ |

*^1^Fisher’s Exact Test (when one or multiple cells had expected frequencies of <5 in a 2 x 2 table); ^2^Chi square test*

**Supplementary Table 3: Effects of potential confounders on the total score of the PACIC***

|  | **B**** | **95%CI** | | **p-value** |
| --- | --- | --- | --- | --- |
|  |  | **Lower** | **Upper** |  |
| Sex  Male  Female | 0.330  - | 0.114  - | 0.545  - | **0.003**  **-** |
| Age  BMI | -0.005  0.019 | -0.016  0.001 | 0.006  0.037 | 0.415  **0.041** |
| Smoking status, n (%)  Never smoked  Ex-smoker  Current smoker | -0.024  -0.103  - | -0.334  -0.395  - | 0.287  0.189  - | 0.880  0.489  - |
| National background  Dutch  1st generation migrant  2nd generation migrant | -0.206  -0.119  - | -0.540  -0.687  - | 0.129  0.449 | 0.229  0.681 |
| Educational level  Low  Medium  High | 0.147  -0.018  - | -0.095  -0.247  - | 0.389  0.212  - | 0.233  0.879  - |
| Diagnosed with Covid19  No  Yes | 0.078  - | -0.143  - | 0.299  - | 0.489  - |
| Diagnosed with COPD  No  Yes | 0.185  - | -0.125  - | 0.496  - | 0.242  - |
| Diagnosed with asthma  No  Yes | -0.073  - | -0.408  - | 0.262  - | 0.670  - |
| Diagnosed with type 2 diabetes  No  Yes | -0.041  - | -0.382  - | 0.300  - | 0.814  - |
| Diagnosed with heart failure  No  Yes | -0.010  - | -0.289  - | 0.269  - | 0.943  - |
| Diagnosed with other diseases  No  Yes | 0.015  - | -0.171  - | 0.201  - | 0.873  - |
| Location of the general practice  Urban  Rural | -0.029  - | -0.291  - | 0.233  - | 0.828  - |
| General practitioner with specialisation in COPD, asthma, type 2 diabetes, or heart failure  No  Yes | -0.205  - | -0.561  - | 0.152  - | 0.260  - |
| Year of graduation managing general practitioner  <10 years ago  >10 years ago | -0.071  - | -0.430  - | 0.288  - | 0.698  - |
| General practice in a health centre  Yes  No | 0.059  - | -0.183  - | 0.300  - | 0.633  - |
| Possibility of consulting a specialist within primary care  Yes  No | -0.112  - | -0.544  - | 0.319  - | 0.609 |

*These effects were established with mixed linear regression of which the intervention effects are reported in Tabel 2. The intra-class correlation (ICC) at 18 months = 0.033; **B = mixed linear regression weight for each potential confounder, corrected for the other variables in the model (other potential confounders, treatment arm, time, and interaction between treatment arm and time). B >0 indicates a positive association with PACIC total score. PACIC = Patient Assessment of Chronic Illness Care.

***Secondary outcomes***

**Supplementary Table 4: Effect of the ABCC-tool on the total score and subdomains of the PACIC at T6, T12, and T18 for type 2 diabetes; observed outcomes and intervention effects as established with mixed linear regression and corrected for potential confounders***

|  | **Score in intervention group, mean (SD); n** | **Score in control group, mean (SD); n** | **B**** | **95%CI** | | **p-value** |
| --- | --- | --- | --- | --- | --- | --- |
|  |  |  |  | **Lower** | **Upper** |  |
| Total score  Baseline  6 months  12 months  18 months | 3.14 (0.78); 150  3.11 (0.78); 130  3.03 (0.86); 115  3.03 (0.87); 80 | 3.17 (0.75); 45  2.81 (0.84); 42  2.66 (0.86); 40  2.69 (0.94); 36 | -  0.351  0.401  0.298 | -  0.078  0.116  -0.021 | -  0.624  0.685  0.617 | -  **0.012**  **0.006**  0.067 |
| Patient activation  Baseline  6 months  12 months  18 months | 3.78 (1.05); 151  3.63 (1.21); 130  3.60 (1.13); 115  3.48 (1.22); 80 | 3.97 (1.13); 45  3.34 (1.17); 42  3.22 (1.14); 40  3.10 (1.14); 36 | -  0.475  0.573  0.668 | -  0.017  0.129  0.257 | -  0.933  1.017  1.080 | -  **0.042**  **0.011**  **0.001** |
| Delivery system support  Baseline  6 months  12 months  18 months | 3.62 (0.75); 151  3.57 (0.76); 130  3.51 (0.80); 114  3.41 (0.80); 80 | 3.65 (0.79); 45  3.32 (0.76); 42  3.10 (0.91); 40  3.13 (0.85); 36 | -  0.310  0.411  0.232 | -  0.021  0.107  -0.109 | -  0.600  0.715  0.572 | -  **0.036**  **0.008**  0.180 |
| Goal setting  Baseline  6 months  12 months  18 months | 2.91 (0.88); 150  2.96 (0.84); 130  2.89 (0.95); 114  2.79 (0.91); 80 | 3.00 (0.91); 45  2.61 (0.96); 42  2.45 (0.88); 40  2.52 (1.05); 36 | -  0.463  0.554  0.282 | -  0.174  0.235  -0.080 | -  0.752  0.872  0.644 | -  **0.002**  **<0.001**  0.127 |
| Problem solving  Baseline  6 months  12 months  18 months | 3.40 (1.08); 150  3.53 (1.11); 130  3.35 (1.13); 115  3.33 (1.13); 80 | 3.41 (1.04); 45  3.10 (1.18); 42  2.82 (1.15); 40  2.88 (1.20); 36 | -  0.449  0.548  0.359 | -  0.042  0.143  -0.070 | -  0.856  0.953  0.787 | -  **0.030**  **0.008**  0.101 |
| Follow-up /coordination  Baseline  6 months  12 months  18 months | 2.46 (0.90); 150  2.34 (0.86); 130  2.32 (0.88);114  2.53 (0.92); 80 | 2.37 (0.83); 45  2.13 (0.90); 42  2.17 (0.89); 40  2.18 (0.96); 36 | -  0.112  0.067  0.138 | -  -0.193  -0.245  -0.232 | -  0.417  0.378  0.509 | -  0.471  0.676  0.464 |

Scale ranges from 1 to 5. *Adjusted for all potential confounders (Supplementary Material 6); **B = mixed linear regression weight for treatment, indicating the estimated difference between intervention and control at 6, 12 or 18 months, corrected for the outcome at baseline and potential confounders. B >0 indicates a higher score in the intervention group. ABCC-tool = Assessment of Burden of Chronic Conditions tool; PACIC = Patient Assessment of Chronic Illness Care.

**Supplementary Table 5: Effect of the ABCC-tool on the EQ-5D-5L at T6, T12, and T18 for the total group; observed outcomes and intervention effects as established with mixed linear regression and corrected for potential confounders***

|  | **Score in intervention group, mean (SD); n** | **Score in control group, mean (SD); n** | **B**** | **95%CI** | | **p-value** |
| --- | --- | --- | --- | --- | --- | --- |
|  |  |  |  | **Lower** | **Upper** |  |
| Index score  Baseline  6 months  12 months  18 months | 0.85 (0.17); 173  0.84 (0.17); 151  0.85 (0.15); 132  0.85 (0.13); 91 | 0.87 (0.14); 59  0.86 (0.17); 54  0.86 (0.16); 50  0.83 (0.16); 43 | -  0.013  0.023  0.041 | -  -0.031  -0.015  -0.001 | -  0.056  0.062  0.083 | -  0.570  0.232  0.057 |
| Visual Analogue Scale  Baseline  6 months  12 months  18 months | 76.66 (14.44); 175  76.76 (14.25); 151  77.53 (13.69); 132  76.70 (14.11); 90 | 77.75 (13.59); 59  77.72 (13.45); 54  78.70 (12.82); 50  75.39 (14.78); 44 | -  0.203  -0.228  0.691 | -  -3.991  -4.004  -4.031 | -  4.397  3.547  5.414 | -  0.924  0.906  0.774 |

Index score ranges from less than 0 (where 0 is the value of a health state equivalent to dead) to 1 (full health); VAS ranges from 0 to 100. *Adjusted for all potential confounders (Supplementary Material 6); **B = mixed linear regression weight for treatment, indicating the estimated difference between intervention and control at 6, 12 or 18 months, corrected for the outcome at baseline and potential confounders. B >0 indicates a higher score in the intervention group. ABCC-tool = Assessment of Burden of Chronic Conditions tool; EQ-5D-5L = EuroQol-5D-5L; VAS = Visual Analogue Scale

**Supplementary Table 6: Effect of the ABCC-tool on the EQ-5D-5L at T6, T12, and T18 for type 2 diabetes; observed outcomes and intervention effects as established with mixed linear regression and corrected for potential confounders***

|  | **Score in intervention group, mean (SD); n** | **Score in control group, mean (SD); n** | **B**** | **95%CI** | | **p-value** |
| --- | --- | --- | --- | --- | --- | --- |
|  |  |  |  | **Lower** | **Upper** |  |
| Index score  Baseline  6 months  12 months  18 months | 0.85 (0.18); 149  0.84 (0.18); 131  0.85 (0.16); 115  0.85 (0.13); 80 | 0.86 (0.14); 45  0.87 (0.14); 42  0.85 (0.16); 40  0.83 (0.16); 35 | -  -0.015  0.014  0.032 | **-**  -0.063  -0.028  -0.013 | **-**  0.034  0.056  0.077 | -  0.551  0.519  0.159 |
| Visual Analogue Scale  Baseline  6 months  12 months  18 months | 77.83 (13.94); 151  76.82 (14.65); 131  77.53 (14.28); 115  77.51 (14.28); 79 | 77.04 (14.67); 45  77.67 (12.25); 42  78.08 (13.27); 40  75.11 (14.66); 36 | -  -1.396  -1.956  -0.071 | -  -6.066  -6.108  -5.376 | -  3.274  2.195  5.235 | -  0.558  0.356  0.379 |

Index score ranges from less than 0 (where 0 is the value of a health state equivalent to dead) to 1 (full health); VAS ranges from 0 to 100. *Adjusted for all potential confounders (Supplementary Material 6); **B = mixed linear regression weight for treatment, indicating the estimated difference between intervention and control at 6, 12 or 18 months, corrected for the outcome at baseline and potential confounders. B >0 indicates a higher score in the intervention group. ABCC-tool = Assessment of Burden of Chronic Conditions tool; EQ-5D-5L = EuroQol-5D-5L; VAS = Visual Analogue Scale

**Supplementary Table 7: Effect of the ABCC-tool on the PAM at T6, T12, and T18 for the total group; observed outcomes and intervention effects as established with mixed linear regression and corrected for potential confounders***

|  | **Score in intervention group, mean (SD); n** | **Score in control group, mean (SD); n** | **B**** | **95%CI** | | **p-value** |
| --- | --- | --- | --- | --- | --- | --- |
|  |  |  |  | **Lower** | **Upper** |  |
| Activation score PAM  Baseline  6 months  12 months  18 months | 64.24 (14.30); 170  65.95 (15.51); 148  67.28 (14.84); 126  66.48 (16.50); 90 | 66.23 (16.12); 57  64.90 (12.96); 53  63.97 (14.88); 48  61.68 (12.64); 44 | -  2.995  4.302  5.768 | -  -1.031  -0.341  0.776 | -  7.022  8.945  10.760 | -  0.145  0.069  **0.024** |

Scale ranges from 0 to 100. *Adjusted for all potential confounders (Supplementary Material 6); **B = mixed linear regression weight for treatment, indicating the estimated difference between intervention and control at 6, 12 or 18 months, corrected for the outcome at baseline and potential confounders. B >0 indicates a higher score in the intervention group. ABCC-tool = Assessment of Burden of Chronic Conditions tool; PAM = Patient Activation Measure

**Supplementary Table 8: Effect of the ABCC-tool on the PAM at T6, T12, and T18 for the type 2 diabetes; observed outcomes and intervention effects as established with mixed linear regression and corrected for potential confounders***

|  | **Score in intervention group, mean (SD); n** | **Score in control group, mean (SD); n** | **B**** | **95%CI** | | **p-value** |
| --- | --- | --- | --- | --- | --- | --- |
|  |  |  |  | **Lower** | **Upper** |  |
| Activation score PAM  Baseline  6 months  12 months  18 months | 63.71 (14.04); 147  65.73 (15.30); 128  67.47 (15.51); 109  66.38 (16.71); 79 | 66.60 (16.20); 44  66.24 (12.61); 41  64.43 (16.01); 39  62.94 (12.91); 36 | -  2.613  4.743  5.218 | **-**  -1.399  -0.289  -0.129 | -  6.625  9.775  10.564 | -  0.202  0.065  0.056 |

Scale ranges from 0 to 100. *Adjusted for all potential confounders (Supplementary Material 6); **B = mixed linear regression weight for treatment, indicating the estimated difference between intervention and control at 6, 12 or 18 months, corrected for the outcome at baseline and potential confounders. B >0 indicates a higher score in the intervention group. ABCC-tool = Assessment of Burden of Chronic Conditions tool; PAM = Patient Activation Measure

**Supplementary Table 9: Effect of the ABCC-tool on the ICECAP-A at T6, T12, and T18 for the total group; observed outcomes and intervention effects as established with mixed linear regression and corrected for potential confounders***

|  | **Score in intervention group, mean (SD); n** | **Score in control group, mean (SD); n** | **B**** | **95%CI** | | **p-value** |
| --- | --- | --- | --- | --- | --- | --- |
|  |  |  |  | **Lower** | **Upper** |  |
| Overall score  Baseline  6 months  12 months  18 months | 0.88 (0.12); 175  0.89 (0.13); 149  0.89 (0.13); 131  0.88 (0.12); 89 | 0.90 (0.11); 59  0.89 (0.13); 54  0.88 (0.12); 49  0.88 (0.12); 44 | -  0.014  0.031  0.024 | -  -0.014  0.000  -0.009 | -  0.042  0.062  0.056 | -  0.320  0.052  0.155 |

Scale ranges from 0 to 1. Adjusted for all potential confounders (Supplementary Material 6); **B = mixed linear regression weight for treatment, indicating the estimated difference between intervention and control at 6, 12 or 18 months, corrected for the outcome at baseline and potential confounders. B >0 indicates a higher score in the intervention group. ABCC-tool = Assessment of Burden of Chronic Conditions tool; ICECAP-A = ICEpop CAPability measure for Adults

**Supplementary Table 10: Effect of the ABCC-tool on the ICECAP-A at T6, T12, and T18 for type 2 diabetes; observed outcomes and intervention effects as established with mixed linear regression and corrected for potential confounders***

|  | **Score in intervention group, mean (SD); n** | **Score in control group, mean (SD); n** | **B**** | **95%CI** | | **p-value** |
| --- | --- | --- | --- | --- | --- | --- |
|  |  |  |  | **Lower** | **Upper** |  |
| Overall score  Baseline  6 months  12 months  18 months | 0.89 (0.13); 151  0.89 (0.13); 129  0.88 (0.14); 114  0.88 (0.13); 78 | 0.90 (0.10); 45  0.90 (0.09); 42  0.88 (0.12); 39  0.89 (0.11); 36 | -  0.002  0.016  0.009 | -  -0.025  -0.018  -0.026 | -  0.029  0.049  0.044 | -  0.883  0.353  0.605 |

Scale ranges from 0 to 1. Adjusted for all potential confounders (Supplementary Material 6); **B = mixed linear regression weight for treatment, indicating the estimated difference between intervention and control at 6, 12 or 18 months, corrected for the outcome at baseline and potential confounders. B >0 indicates a higher score in the intervention group. ABCC-tool = Assessment of Burden of Chronic Conditions tool; ICECAP-A = ICEpop CAPability measure for Adults

***Sensitivity analyses: selection potential confounders***

**Supplementary Table 11: Effect of the ABCC-tool on the total score and subdomains of the PACIC at T6, T12, and T18 for the total group; observed outcomes and intervention effects as established with mixed linear regression and corrected for selected potential confounders***

|  | **Score in intervention group, mean (SD); n** | **Score in control group, mean (SD); n** | **B**** | **95%CI** | | **p-value** |
| --- | --- | --- | --- | --- | --- | --- |
|  |  |  |  | **Lower** | **Upper** |  |
| Total score  Baseline  6 months  12 months  18 months | 3.06 (0.82); 174  3.10 (0.79); 150  2.98 (0.90); 132  3.05 (0.89); 91 | 3.17 (0.81); 59  2.83 (0.85); 54  2.68 (0.84); 50  2.73 (0.97); 44 | -  0.386  0.405  0.391 | -  0.130  0.147  0.092 | -  0.643  0.663  0.689 | **0.003**  **0.002**  **0.010** |
| Patient activation  Baseline  6 months  12 months  18 months | 3.69 (1.13); 175  3.63 (1.20); 150  3.53 (1.16); 132  3.53 (1.22); 91 | 3.95 (1.10); 59  3.36 (1.17); 54  3.22 (1.12); 50  3.16 (1.16); 44 | -  0.521  0.560  0.735 | -  0.106  0.160  0.339 | -  0.936  0.960  1.131 | -  **0.014**  **0.006**  **<0.001** |
| Delivery system support  Baseline  6 months  12 months  18 months | 3.54 (0.82); 175  3.56 (0.76); 150  3.44 (0.86); 131  3.44 (0.81); 91 | 3.69 (0.84); 59  3.31 (0.81); 54  3.12 (0.91); 50  3.17 (0.86); 44 | -  0.397  0.417  0.364 | -  0.136  0.152  0.047 | -  0.658  0.682  0.681 | -  **0.003**  **0.002**  **0.024** |
| Goal setting  Baseline  6 months  12 months  18 months | 2.84 (0.90); 174  2.95 (0.86); 150  2.82 (1.00); 131  2.80 (0.93); 91 | 2.97 (0.97); 59  2.63 (0.94); 54  2.46 (0.87); 50  2.59 (1.10); 44 | -  0.459  0.510  0.306 | -  0.181  0.222  -0.036 | -  0.736  0.798  0.647 | -  **0.001**  **<0.001**  0.079 |
| Problem solving  Baseline  6 months  12 months  18 months | 3.33 (1.09); 174  3.51 (1.10); 150  3.29 (1.17); 132  3.33 (1.17); 91 | 3.32 (1.16); 59  3.09 (1.16); 54  2.86 (1.13); 50  2.90 (1.25); 44 | -  0.400  0.433  0.419 | -  0.027  0.064  0.026 | -  0.773  0.802  0.813 | -  **0.035**  **0.022**  **0.037** |
| Follow-up /coordination  Baseline  6 months  12 months  18 months | 2.37 (0.91); 174  2.33 (0.87); 150  2.31 (0.92); 131  2.54 (0.95); 91 | 2.48 (1.00); 59  2.21 (0.89); 54  2.16 (0.88); 50  2.23 (1.00); 44 | -  0.220  0.228  0.294 | -  -0.062  -0.061  -0.042 | -  0.501  0.517  0.629 | -  0.126  0.122  0.086 |

Scale ranges from 1 to 5. *Adjusted for: sex (man/ woman) and body mass index (kg/m2; continuous); **B = mixed linear regression weight for treatment, indicating the estimated difference between intervention and control at 6, 12 or 18 months, corrected for the outcome at baseline and potential confounders. B >0 indicates a higher score in the intervention group. ABCC-tool = Assessment of Burden of Chronic Conditions tool; PACIC = Patient Assessment of Chronic Illness Care.

**Supplementary Table 12: Effect of the ABCC-tool on the EQ-5D-5L at T6, T12, and T18 for the total group; observed outcomes and intervention effects as established with mixed linear regression and corrected for selected potential confounders**

|  | **Score in intervention group, mean (SD); n** | **Score in control group, mean (SD); n** | **B***** | **95%CI** | | **p-value** |
| --- | --- | --- | --- | --- | --- | --- |
|  |  |  |  | **Lower** | **Upper** |  |
| Index score*  Baseline  6 months  12 months  18 months | 0.85 (0.17); 173  0.84 (0.17); 151  0.85 (0.15); 132  0.85 (0.13); 91 | 0.87 (0.14); 59  0.86 (0.17); 54  0.86 (0.16); 50  0.83 (0.16); 43 | -  0.013  0.024  0.041 | -  -0.031  -0.014  -0.001 | -  0.056  0.063  0.083 | -  0.563  0.220  0.056 |
| Visual Analogue Scale**  Baseline  6 months  12 months  18 months | 76.66 (14.44); 175  76.76 (14.25); 151  77.53 (13.69); 132  76.70 (14.11); 90 | 77.75 (13.59); 59  77.72 (13.45); 54  78.70 (12.82); 50  75.39 (14.78); 44 | -  0.282  -0.205  0.630 | -  -3.904  -3.980  -4.094 | -  4.469  3.570  5.353 | -  0.895  0.915  0.794 |

Index score ranges from less than 0 (where 0 is the value of a health state equivalent to dead) to 1 (full health); VAS ranges from 0 to 100. *Adjusted for body mass index (kg/m2; continuous), asthma (yes/no), other disease (yes/no), diagnosed COVID-19 (yes/no), and age (years; continuous); ** adjusted for body mass index (kg/m2; continuous), COPD (yes/no), heart failure (yes/no), and diagnosed COVID-19 (yes/no); ***B = mixed linear regression weight for treatment, indicating the estimated difference between intervention and control at 6, 12 or 18 months, corrected for the outcome at baseline and potential confounders. B >0 indicates a higher score in the intervention group. ABCC-tool = Assessment of Burden of Chronic Conditions tool; EQ-5D-5L = EuroQol-5D-5L; VAS = Visual Analogue Scale

**Supplementary Table 13: Effect of the ABCC-tool on the PAM at T6, T12, and T18 for the total group; observed outcomes and intervention effects as established with mixed linear regression and corrected for selected potential confounders***

|  | **Score in intervention group, mean (SD); n** | **Score in control group, mean (SD); n** | **B**** | **95%CI** | | **p-value** |
| --- | --- | --- | --- | --- | --- | --- |
|  |  |  |  | **Lower** | **Upper** |  |
| Activation score PAM  Baseline  6 months  12 months  18 months | 64.24 (14.30); 170  65.95 (15.51); 148  67.28 (14.84); 126  66.48 (16.50); 90 | 66.23 (16.12); 57  64.90 (12.96); 53  63.97 (14.88); 48  61.68 (12.64); 44 | -  3.213  4.423  5.849 | -  -0.808  -0.221  0.870 | -  7.233  9.067  10.829 | -  0.117  0.062  **0.021** |

Scale ranges from 0 to 100. *Adjusted for body mass index (kg/m2; continuous); **B = mixed linear regression weight for treatment, indicating the estimated difference between intervention and control at 6, 12 or 18 months, corrected for the outcome at baseline and potential confounders. B >0 indicates a higher score in the intervention group. ABCC-tool = Assessment of Burden of Chronic Conditions tool; PAM = Patient Activation Measure

**Supplementary Table 14: Effect of the ABCC-tool on the ICECAP-A at T6, T12, and T18 for the total group; observed outcomes and intervention effects as established with mixed linear regression and corrected for selected potential confounders***

|  | **Score in intervention group, mean (SD); n** | **Score in control group, mean (SD); n** | **B**** | **95%CI** | | **p-value** |
| --- | --- | --- | --- | --- | --- | --- |
|  |  |  |  | **Lower** | **Upper** |  |
| Overall score  Baseline  6 months  12 months  18 months | 0.88 (0.12); 175  0.89 (0.13); 149  0.89 (0.13); 131  0.88 (0.12); 89 | 0.90 (0.11); 59  0.89 (0.13); 54  0.88 (0.12); 49  0.88 (0.12); 44 | -  0.015  0.032  0.024 | -  -0.013  0.001  -0.009 | -  0.042  0.062  0.056 | -  0.302  **0.044**  0.157 |

Scale ranges from 0 to 1. *Adjusted for age (years; continuous), asthma (yes/no), and other disease (yes/no); **B = mixed linear regression weight for treatment, indicating the estimated difference between intervention and control at 6, 12 or 18 months, corrected for the outcome at baseline and potential confounders. B >0 indicates a higher score in the intervention group. ABCC-tool = Assessment of Burden of Chronic Conditions tool; ICECAP-A = ICEpop CAPability measure for Adults

***Sensitivity analyses: per protocol***

**Supplementary Table 15: Effect of the ABCC-tool on the total score and subdomains of the PACIC at T6, T12, and T18 for total group, per protocol; observed outcomes and intervention effects as established with mixed linear regression and corrected for potential confounders***

|  | **Score in intervention group, mean (SD); n** | **Score in control group, mean (SD); n** | **B**** | **95%CI** | | **p-value** |
| --- | --- | --- | --- | --- | --- | --- |
|  |  |  |  | **Lower** | **Upper** |  |
| Total score  Baseline  6 months  12 months  18 months | 3.12 (0.84); 128  3.13 (0.83); 113  3.04 (0.94); 99  3.12 (0.95); 65 | 3.17 (0.81); 59  2.83 (0.85); 54  2.68 (0.84); 50  2.73 (0.97); 44 | -  0.363  0.413  0.382 | -  0.099  0.144  0.060 | -  0.627  0.683  0.705 | -  **0.007**  **0.003**  **0.020** |
| Patient activation  Baseline  6 months  12 months  18 months | 3.79 (1.12); 129  3.66 (1.24); 113  3.57 (1.21); 99  3.57 (1.28); 65 | 3.95 (1.10); 59  3.36 (1.17); 54  3.22 (1.12); 50  3.16 (1.16); 44 | -  0.468  0.536  0.677 | -  0.021  0.111  0.254 | -  0.914  0.960  1.100 | -  **0.040**  **0.013**  **0.002** |
| Delivery system design/decision support  Baseline  6 months  12 months  18 months | 3.59 (0.84); 129  3.61 (0.76); 113  3.53 (0.87); 98  3.50 (0.88); 65 | 3.69 (0.84); 59  3.31 (0.81); 54  3.12 (0.91); 50  3.17 (0.86); 44 | -  0.416  0.479  0.358 | -  0.151  0.198  0.012 | -  0.680  0.760  0.705 | -  **0.002**  **<0.001**  **0.043** |
| Goal setting/tailoring  Baseline  6 months  12 months  18 months | 2.89 (0.92); 128  2.97 (0.91); 113  2.91 (1.03); 98  2.91 (0.97); 65 | 2.97 (0.97); 59  2.63 (0.94); 54  2.46 (0.87); 50  2.59 (1.10); 44 | -  0.421  0.535  0.309 | -  0.140  0.232  -0.063 | -  0.702  0.838  0.680 | -  **0.003**  **<0.001**  0.103 |
| Problem solving/contextual  Baseline  6 months  12 months  18 months | 3.43 (1.11); 128  3.55 (1.11); 113  3.35 (1.20); 99  3.40 (1.19); 65 | 3.32 (1.16); 59  3.09 (1.16); 54  2.86 (1.13); 50  2.90 (1.25); 44 | -  0.367  0.402  0.376 | -  -0.023  0.013  -0.041 | -  0.757  0.791  0.793 | -  0.065  **0.043**  0.077 |
| Follow-up/coordination  Baseline  6 months  12 months  18 months | 2.40 (0.94); 128  2.36 (0.93); 113  2.32 (0.97); 98  2.60 (0.97); 65 | 2.48 (1.00); 59  2.11 (0.89); 54  2.16 (0.88); 50  2.23 (1.00); 44 | -  0.211  0.229  0.304 | -  -0.081  -0.065  -0.050 | -  0.503  0.523  0.658 | -  0.157  0.126  0.093 |

Scale ranges from 1 to 5. *Adjusted for all potential confounders (Supplementary Material 6); **B = mixed linear regression weight for treatment, indicating the estimated difference between intervention and control at 6, 12 or 18 months, corrected for the outcome at baseline and potential confounders. B >0 indicates a higher score in the intervention group. ABCC-tool = Assessment of Burden of Chronic Conditions tool; PACIC = Patient Assessment of Chronic Illness Care.

**Supplementary Table 16: Effect of the ABCC-tool on the EQ-5D-5L at T6, T12, and T18 for total group, per protocol; observed outcomes and intervention effects as established with mixed linear regression and corrected for potential confounders***

|  | **Score in intervention group, mean (SD); n** | **Score in control group, mean (SD); n** | **B**** | **95%CI** | | **p-value** |
| --- | --- | --- | --- | --- | --- | --- |
|  |  |  |  | **Lower** | **Upper** |  |
| Index score  Baseline  6 months  12 months  18 months | 0.84 (0.17); 130  0.84 (0.17); 114  0.86 (0.13); 100  0.86 (0.11); 66 | 0.87 (0.14); 59  0.86 (0.17); 54  0.86 (0.16); 50  0.83 (0.16); 43 | -  0.018  0.035  0.051 | -  -0.028  -0.006  0.008 | -  0.065  0.076  0.095 | -  0.439  0.096  **0.020** |
| Visual Analogue Scale  Baseline  6 months  12 months  18 months | 76.34 (14.43); 129  76.53 (14.48); 114  77.83 (12.82); 99  77.28 (13.79); 64 | 77.75 (13.59); 59  77.72 (13.45); 54  78.70 (12.82); 50  75.39 (14.78); 44 | -  0.607  0.464  1.172 | -  -3.895  -3.460  -3.773 | -  5.110  4.388  6.118 | -  0.791  0.817  0.642 |

Index score ranges from less than 0 (where 0 is the value of a health state equivalent to dead) to 1 (full health); VAS ranges from 0 to 100. *Adjusted for all potential confounders (Supplementary Material 6); **B = mixed linear regression weight for treatment, indicating the estimated difference between intervention and control at 6, 12 or 18 months, corrected for the outcome at baseline and potential confounders. B >0 indicates a higher score in the intervention group. ABCC-tool = Assessment of Burden of Chronic Conditions tool; EQ-5D-5L = EuroQol-5D-5L; VAS = Visual Analogue Scale

**Supplementary Table 17: Effect of the ABCC-tool on the PAM at T6, T12, and T18 for the total group, per protocol; observed outcomes and intervention effects as established with mixed linear regression and corrected for potential confounders***

|  | **Score in intervention group, mean (SD); n** | **Score in control group, mean (SD); n** | **B**** | **95%CI** | | **p-value** |
| --- | --- | --- | --- | --- | --- | --- |
|  |  |  |  | **Lower** | **Upper** |  |
| Activation score PAM  Baseline  6 months  12 months  18 months | 64.48 (14.05); 127  65.69 (15.76); 112  66.61 (14.24); 96  66.18 (16.56); 64 | 66.22 (16.12); 57  64.90 (12.96); 53  63.97 (14.88); 48  61.68 (12.64); 44 | -  2.528  3.592  5.001 | -  -1.793  -1.240  -0.352 | -  6.850  8.423  10.353 | -  0.251  0.145  0.067 |

Scale ranges from 0 to 100. *Adjusted for all potential confounders (Supplementary Material 6); **B = mixed linear regression weight for treatment, indicating the estimated difference between intervention and control at 6, 12 or 18 months, corrected for the outcome at baseline and potential confounders. B >0 indicates a higher score in the intervention group. ABCC-tool = Assessment of Burden of Chronic Conditions tool; PAM = Patient Activation Measure

**Supplementary Table 18: Effect of the ABCC-tool on the ICECAP-A at T6, T12, and T18 for the total group, per protocol; observed outcomes and intervention effects as established with mixed linear regression and corrected for potential confounders***

|  | **Score in intervention group, mean (SD); n** | **Score in control group, mean (SD); n** | **B**** | **95%CI** | | **p-value** |
| --- | --- | --- | --- | --- | --- | --- |
|  |  |  |  | **Lower** | **Upper** |  |
| Overall score  Baseline  6 months  12 months  18 months | 0.88 (0.13); 130  0.88 (0.14); 112  0.88 (0.13); 99  0.89 (0.11); 64 | 0.90 (0.11); 59  0.89 (0.13); 54  0.88 (0.12); 49  0.88 (0.12); 44 | -  0.015  0.037  0.035 | -  -0.015  0.002  0.004 | -  0.045  0.072  0.065 | -  0.326  **0.037**  **0.024** |

Scale ranges from 0 to 1. Adjusted for all potential confounders (Supplementary Material 6); **B = mixed linear regression weight for treatment, indicating the estimated difference between intervention and control at 6, 12 or 18 months, corrected for the outcome at baseline and potential confounders. B >0 indicates a higher score in the intervention group. ABCC-tool = Assessment of Burden of Chronic Conditions tool; ICECAP-A = ICEpop CAPability measure for Adults

***Sensitivity analyses: including physical consultations***

**Supplementary Table 19: Effect of treatment (ABCC-tool) on the total score and subdomains of the PACIC at T6, T12, and T18 for the total group; observed outcomes and intervention effects as established with mixed linear regression and corrected for potential confounders, including physical consultations***

|  | **Score in intervention group, mean (SD); n** | **Score in control group, mean (SD); n** | **B**** | **95%CI** | | **p-value** |
| --- | --- | --- | --- | --- | --- | --- |
|  |  |  |  | **Lower** | **Upper** |  |
| Total score  Baseline  6 months  12 months  18 months | 3.06 (0.82); 174  3.10 (0.79); 150  2.98 (0.90); 132  3.05 (0.89); 91 | 3.17 (0.81); 59  2.83 (0.85); 54  2.68 (0.84); 50  2.73 (0.97); 44 | -  0.365  0.392  0.369 | -  0.105  0.122  0.084 | -  0.624  0.662  0.653 | -  **0.006**  **0.004**  **0.011** |
| Patient activation  Baseline  6 months  12 months  18 months | 3.69 (1.13); 175  3.63 (1.20); 150  3.53 (1.16); 132  3.53 (1.22); 91 | 3.95 (1.10); 59  3.36 (1.17); 54  3.22 (1.12); 50  3.16 (1.16); 44 | -  0.474  0.517  0.737 | -  0.046  0.106  0.343 | -  0.903  0.928  1.131 | -  **0.030**  **0.014**  **<0.001** |
| Delivery system support  Baseline  6 months  12 months  18 months | 3.54 (0.82); 175  3.56 (0.76); 150  3.44 (0.86); 131  3.44 (0.81); 91 | 3.69 (0.84); 59  3.31 (0.81); 54  3.12 (0.91); 50  3.17 (0.86); 44 | -  0.426  0.421  0.345 | -  0.158  0.153  0.042 | -  0.694  0.699  0.649 | -  **0.002**  **0.003**  **0.026** |
| Goal setting  Baseline  6 months  12 months  18 months | 2.84 (0.90); 174  2.95 (0.86); 150  2.82 (1.00); 131  2.80 (0.93); 91 | 2.97 (0.97); 59  2.63 (0.94); 54  2.46 (0.87); 50  2.59 (1.10); 44 | -  0.451  0.482  0.271 | -  0.167  0.178  -0.054 | -  0.734  0.787  0.595 | -  **0.002**  **0.002**  0.102 |
| Problem solving  Baseline  6 months  12 months  18 months | 3.33 (1.09); 174  3.51 (1.10); 150  3.29 (1.17); 132  3.33 (1.17); 91 | 3.32 (1.16); 59  3.09 (1.16); 54  2.86 (1.13); 50  2.90 (1.25); 44 | -  0.361  0.444  0.397 | -  -0.018  0.058  0.014 | -  0.741  0.830  0.781 | -  0.062  **0.024**  **0.042** |
| Follow-up /coordination  Baseline  6 months  12 months  18 months | 2.37 (0.91); 174  2.33 (0.87); 150  2.31 (0.92); 131  2.54 (0.95); 91 | 2.48 (1.00); 59  2.21 (0.89); 54  2.16 (0.88); 50  2.23 (1.00); 44 | -  0.194  0.221  0.209 | -  -0.097  -0.076  -0.115 | -  0.484  0.517  0.533 | -  0.192  0.145  0.205 |

Scale ranges from 1 to 5. *Adjusted for all potential confounders (Supplementary Material 6) and number of physical consultations in the previous six months (continuous); **B = mixed linear regression weight for treatment, indicating the estimated difference between intervention and control at 6, 12 or 18 months, corrected for the outcome at baseline and potential confounders. B >0 indicates a higher score in the intervention group. ABCC-tool = Assessment of Burden of Chronic Conditions tool; PACIC = Patient Assessment of Chronic Illness Care.

**Supplementary Table 20: Effect of the ABCC-tool on the EQ-5D-5L at T6, T12, and T18 for the total group; observed outcomes and intervention effects as established with mixed linear regression and corrected for potential confounders, including physical consultations***

|  | **Score in intervention group, mean (SD); n** | **Score in control group, mean (SD); n** | **B**** | **95%CI** | | **p-value** |
| --- | --- | --- | --- | --- | --- | --- |
|  |  |  |  | **Lower** | **Upper** |  |
| Index score  Baseline  6 months  12 months  18 months | 0.85 (0.17); 173  0.84 (0.17); 151  0.85 (0.15); 132  0.85 (0.13); 91 | 0.87 (0.14); 59  0.86 (0.17); 54  0.86 (0.16); 50  0.83 (0.16); 43 | -  0.028  0.040  0.052 | -  -0.021  -0.004  0.010 | -  0.077  0.083  0.094 | -  0.266  0.072  **0.016** |
| Visual Analogue Scale  Baseline  6 months  12 months  18 months | 76.66 (14.44); 175  76.76 (14.25); 151  77.53 (13.69); 132  76.70 (14.11); 90 | 77.75 (13.59); 59  77.72 (13.45); 54  78.70 (12.82); 50  75.39 (14.78); 44 | -  2.236  0.522  1.995 | -  -2.131  -3.276  -2.415 | -  6.604  4.319  6.405 | -  0.316  0.788  0.375 |

Index score ranges from less than 0 (where 0 is the value of a health state equivalent to dead) to 1 (full health); VAS ranges from 0 to 100. *Adjusted for all potential confounders (Supplementary Material 6) and number of physical consultations in the previous six months (continuous); **B = mixed linear regression weight for treatment, indicating the estimated difference between intervention and control at 6, 12 or 18 months, corrected for the outcome at baseline and potential confounders. B >0 indicates a higher score in the intervention group. ABCC-tool = Assessment of Burden of Chronic Conditions tool; EQ-5D-5L = EuroQol-5D-5L; VAS = Visual Analogue Scale

**Supplementary Table 21: Effect of the ABCC-tool on the PAM at T6, T12, and T18 for the total group; observed outcomes and intervention effects as established with mixed linear regression and corrected for potential confounders, including physical consultations***

|  | **Score in intervention group, mean (SD); n** | **Score in control group, mean (SD); n** | **B**** | **95%CI** | | **p-value** |
| --- | --- | --- | --- | --- | --- | --- |
|  |  |  |  | **Lower** | **Upper** |  |
| Activation score PAM  Baseline  6 months  12 months  18 months | 64.24 (14.30); 170  65.95 (15.51); 148  67.28 (14.84); 126  66.48 (16.50); 90 | 66.23 (16.12); 57  64.90 (12.96); 53  63.97 (14.88); 48  61.68 (12.64); 44 | -  3.032  4.057  5.353 | -  -1.153  -0.728  0.680 | -  7.218  8.842  10.026 | -  0.156  0.097  **0.025** |

Scale ranges from 0 to 100. *Adjusted for all potential confounders (Supplementary Material 6) and number of physical consultations in the previous six months (continuous); **B = mixed linear regression weight for treatment, indicating the estimated difference between intervention and control at 6, 12 or 18 months, corrected for the outcome at baseline and potential confounders. B >0 indicates a higher score in the intervention group. ABCC-tool = Assessment of Burden of Chronic Conditions tool; PAM = Patient Activation Measure

**Supplementary Table 22: Effect of the ABCC-tool on the ICECAP-A at T6, T12, and T18 for the total group; observed outcomes and intervention effects as established with mixed linear regression and corrected for potential confounders, including physical consultations***

|  | **Score in intervention group, mean (SD); n** | **Score in control group, mean (SD); n** | **B**** | **95%CI** | | **p-value** |
| --- | --- | --- | --- | --- | --- | --- |
|  |  |  |  | **Lower** | **Upper** |  |
| Overall score  Baseline  6 months  12 months  18 months | 0.88 (0.12); 175  0.89 (0.13); 149  0.89 (0.13); 131  0.88 (0.12); 89 | 0.90 (0.11); 59  0.89 (0.13); 54  0.88 (0.12); 49  0.88 (0.12); 44 | -  0.017  0.026  0.017 | -  -0.014  -0.005  -0.015 | -  0.047  0.058  0.050 | -  0.289  0.105  0.297 |

Scale ranges from 0 to 1. *Adjusted for all potential confounders (Supplementary Material 6) and number of physical consultations in the previous six months (continuous); **B = mixed linear regression weight for treatment, indicating the estimated difference between intervention and control at 6, 12 or 18 months, corrected for the outcome at baseline and potential confounders. B >0 indicates a higher score in the intervention group. ABCC-tool = Assessment of Burden of Chronic Conditions tool; ICECAP-A = ICEpop CAPability measure for Adults

**Supplementary Figures**

Supplementary Figure 1: Mean change in PAM scores at 6, 12, and 18-month follow-up compared with baseline. The whiskers show the 95% confidence intervals.

| **Inputs** |  | **Activities** |  | **Outputs** |  | **Outcomes**  We expect the program to: |  | **Impact**  The desired long-term impacts of the program are to: |
| --- | --- | --- | --- | --- | --- | --- | --- | --- |
|  |  |  |  |  |  |  |  |  |
| ABCC-tool in information system |  | Train healthcare providers to use the ABCC-tool |  | Number of times the ABCC-questionnaire was completed |  | Improve insight in burden of disease |  | Promote healthy lifestyles, and increase patients’ activation and self-management |
|  |  |  |  |  |  |  |  |  |
| Short explainer film & poster |  | Recruit patients who are willing to use the ABCC-tool |  | Number of times a personalised goal was formulated |  | Increase shared decision making |  | Improve quality of life |
|  |  |  |  |  |  |  |  |  |
| Healthcare providers to implement the ABCC-tool |  | Use the ABCC-tool in consultations between healthcare providers and patients |  |  |  | Improve perceived quality of care |  | Improve patients’ ‘capabilities’ |
|  |  |  |  |  |  |  |  |  |

Supplementary Figure 2: Logic model of the ABCC-tool

Supplementary Figure 3: Completed baseline questionnaires per month

Start COVID-19 measures

Supplementary Figure 4: Number of general practices included in the study per month

**References**

1. Glasgow RE, Wagner EH, Schaefer J, Mahoney LD, Reid RJ, Greene SM. Development and validation of the patient assessment of chronic illness care (PACIC). Med Care. 2005;43(5):436-44.

2. Wensing M, van Lieshout J, Jung HP, Hermsen J, Rosemann T. The Patients Assessment Chronic Illness Care (PACIC) questionnaire in The Netherlands: a validation study in rural general practice. BMC Health Serv Res. 2008;8(1):182.

3. Herdman M, Gudex C, Lloyd A, Janssen M, Kind P, Parkin D, et al. Development and preliminary testing of the new five-level version of EQ-5D (EQ-5D-5L). Qual Life Res. 2011;20(10):1727-36.

4. Feng Y-S, Kohlmann T, Janssen MF, Buchholz I. Psychometric properties of the EQ-5D-5L: a systematic review of the literature. Qual Life Res. 2021;30(3):647-73.

5. Rademakers J, Nijman J, van der Hoek L, Heijmans M, Rijken M. Measuring patient activation in The Netherlands: translation and validation of the American short form Patient Activation Measure (PAM13). BMC Public Health. 2012;12(1):577.

6. Hibbard JH, Mahoney ER, Stockard J, Tusler M. Development and testing of a short form of the patient activation measure. Health Serv Res. 2005;40(6p1):1918-30.

7. Rohrbach PJ, Dingemans AE, Essers BA, Van Furth EF, Spinhoven P, Groothuis-Oudshoorn CG, et al. The ICECAP-A instrument for capabilities: assessment of construct validity and test–retest reliability in a general Dutch population. Qual Life Res. 2022;31(3):687-96.

8. Boudewijns EA, Claessens D, Joore M, Keijsers LC, Van Schayck OC, Winkens B, et al. Effectiveness and cost-effectiveness of the Assessment of Burden of Chronic Conditions (ABCC) tool in patients with COPD, asthma, diabetes mellitus type 2 and heart failure: protocol for a pragmatic clustered quasi-experimental study. BMJ open. 2020;10(11):e037693.

9. Rohrbach PJ, Dingemans AE, Groothuis-Oudshoorn CG, Van Til JA, Essers BA, Van Furth EF, et al. The ICEpop Capability Measure for Adults Instrument for Capabilities: Development of a Tariff for the Dutch General Population. Value Health 2022;25(1):125-32.
